# Sphingolipid composition of circulating extracellular vesicles after myocardial ischemia

**DOI:** 10.1038/s41598-020-73411-7

**Published:** 2020-09-30

**Authors:** J. Burrello, V. Biemmi, M. Dei Cas, M. Amongero, S. Bolis, E. Lazzarini, S. Bollini, G. Vassalli, R. Paroni, L. Barile

**Affiliations:** 1 Laboratory for Cardiovascular Theranostics, Cardiocentro Ticino Foundation, Via Tesserete 48, 6900 Lugano, Switzerland; 2 Faculty of Biomedical Sciences, Università della Svizzera Italiana, Lugano, Switzerland; 3 Department of Health Sciences, Università degli Studi di Milano, Milan, Italy; 4 Department of Mathematical Sciences G. L. Lagrange, Polytechnic University of Torino, Torino, Italy; 5 Regenerative Medicine Laboratory, Dept. of Experimental Medicine (DIMES), University of Genova, Genova, Italy; 6 Laboratory of Cellular and Molecular Cardiology, Cardiocentro Ticino Foundation, Lugano, Switzerland; 7 Institute of Life Science, Scuola Superiore Sant’Anna, Pisa, Italy

**Keywords:** Cardiovascular biology, Sphingolipids

## Abstract

Sphingolipids are structural components of cell membrane, displaying several functions in cell signalling. Extracellular vesicles (EV) are lipid bilayer membrane nanoparticle and their lipid composition may be different from parental cells, with a significant enrichment in sphingolipid species, especially in pathological conditions. We aimed at optimizing EV isolation from plasma and describing the differential lipid content of EV, as compared to whole plasma. As pilot study, we evaluated the diagnostic potential of lipidomic signature of circulating EV in patients with a diagnosis of ST-segment-elevation myocardial infarction (STEMI). STEMI patients were evaluated before reperfusion and 24-h after primary percutaneous coronary intervention. Twenty sphingolipid species were quantified by liquid-chromatography tandem-mass-spectrometry. EV-ceramides, -dihydroceramides, and -sphingomyelins increased in STEMI vs*.* matched controls and decreased after reperfusion. Their levels correlated to hs-troponin, leucocyte count, and ejection fraction. Plasma sphingolipids levels were 500-to-700-fold higher as compared to EV content; nevertheless, only sphingomyelins differed in STEMI *vs.* control patients. Different sphingolipid species were enriched in EV and their linear combination by machine learning algorithms accurately classified STEMI patients at pre-PCI evaluation. In conclusion, EV lipid signature discriminates STEMI patients. These findings may contribute to the identification of novel biomarkers and signaling mechanisms related to cardiac ischemia.

## Introduction

There is growing evidence that circulating lipids can be considered biomarkers for multiple disease^1,2^. Blood levels of sphingolipids, including ceramides, dihydroceramides, and sphingomyelin, have been associated to coronary artery disease (CAD)^3^. As a matter of facts, circulating sphingolipids have been evaluated in subjects with acute myocardial infarction (MI) given their diagnostic potential^4–6^. Likewise, they can also provide relevant prognostic power in predicting long term outcomes, such as cardiovascular death, recurrence of major cardiovascular events, or incidence of post-MI heart failure^1,7–9^. Lipids per se have biological active role during the acute phase of myocardial ischemia, with the occlusion of coronary arteries inducing metabolic shift towards anaerobic glycolysis, production of lactate and hydrogen ions, acidosis and activation of fatty acid energy metabolism^10,11^. The ischemic process also leads to membrane depolarization, with activation of voltage-dependent calcium-channels, increase of intracellular calcium concentration, and activation of calcium-dependent lipases and phospholipases^5^. Enzymes such as ceramidase and sphingomyelinase responsible for catalyzing the metabolism of cell membrane structural lipids (ceramide and sphingomyelin respectively) contribute to membrane disruption and cardiomyocytes apoptosis^5,10^. In light of such evidences, these processes may affect the blood lipidomic profile in patient experiencing myocardial injury.

While the large majority of lipids within plasma and/or serum are associated into lipoproteins, membrane-enveloped, cell-released extracellular vesicles (EV) can also contribute to the lipid composition of blood. Depending on cellular origin of donor cells, glycosphingolipid, ceramide, sphingomyelin and amino-phospholipids are the most enriched lipids in EV^12,13^.

On top of the apoptotic bodies resulting from subcellular fractionation of cells undergoing apoptosis, which are rapidly cleared by macrophages, circulating EV include different populations of secreted vesicles such as exosomes, microvesicles, and ectosomes. Based on biogenesis, exosomes are EV of endosomal origin with 30- to 100-nm diameter size. During endocytic internalization, the inward budding of the plasma membrane gives rise to early endosome followed by a second membrane inversion process of the endosomal limiting membrane leading to the biogenesis of intraluminal vesicles and formation of multivesicular bodies (MVB)^13^. The MVB as endosomal intermediate compartment can either fuse with the plasma membrane, with release of exosomes into the extracellular space, or it can direct endocytosed particles to lysosomal degradation. Microvesicles and ectosomes (ranging in size from 100 to 300 nm up to 1 μm), have been referred to as shedding vesicles released by direct budding of cell membrane^14^. Although both processes, MVB biogenesis and membrane budding, results in topological similarity between donor cell and released vesicles membranes, major differences exist in the lipid composition of such released vesicles when compared with whole cell lipid extract of parental cells, thus demonstrating the existence of specific mechanisms of lipid sorting into EV^13^. Cells forming tissues finely tune lipid composition into vesicle membrane in response to specific stress, which can be further mirrored by circulating EV lipidome. Several studies have reported the differential enrichment of lipid classes from cells to EV from in vitro cultured cells, including prostate cancer cell line^12^, human B-cells^15^, dendritic cells^16^ and Oli-neu cells^17^. Indeed they have shown as cell density and culture conditions may affect the membrane inner and outer leaflet lipid distribution in both cells and secreted EV^18,19^. Moreover, is generally accepted that beyond their structural role as membrane components, lipids play functional role as mediator of EV-based intercellular communications^14,20^. Hence, lipid profile of EV can be thought as relevant potential biomarkers^2^. Despite this considerations, only few studies have assessed how the pathophysiological state of subjects can affect the content of lipids in EV derived from body fluids^2,21^.

One relevant aspect is represented by the challenge of obtaining a pure preparation of EV avoiding lipoparticle remnants^22^. Indeed, the heterogeneous fractions obtained when processing biological fluids are unlikely to provide bona fide assessment of the EV populations^2^. Different isolation protocols have been used to isolate EV from body fluids. Beside the well-known ultracentrifugation (UC) procedure, which is based on serial centrifugation steps followed by ultra-centrifugation at 110,000×*g*, additional methods have been recently implemented, including immunoaffinity, size-exclusion or affinity chromatography^23,24^. Progress has been made in obtaining lipoprotein- and albumin-free plasma EV via an iodixanol density gradient combined to bind elute chromatography (BEC)^25^, such technique is affected by limited yield^25^. In this study we employed UC isolation protocol to obtain plasma-derived EV in order to guarantee specificity without compromising yield^26^, while further optimizing the reduction of cross-contamination, as previously reported^27,28^.

Once established, such protocol has been used to isolate EV from plasma of patients with a diagnosis of ST-segment elevation myocardial infarction (STEMI) versus matched controls. Subsequently, by using liquid chromatography tandem mass spectrometry (LC–MS/MS), we assessed differential lipidomic analysis of whole plasma versus isolated EV. STEMI represents the most severe phenotype of CAD, associated to high mortality. We focused on these patients as they exhibit a clear phenotype that is diagnosed by increased plasma levels of troponin, and specific electrocardiogram changes (elevation of ST segment)^29^. Therefore, here we provided a “proof-of-concept” study based on relevant change in the lipid profile of circulating EV of STEMI patients. By using standardized, fully quantitative and reproducible targeted lipidomic analysis technique^30^, we focused on the most relevant species as biomarker STEMI, such as ceramides, dihydroceramides, and sphingomyelins. Finally, by using machine learning algorithms, we evaluated the diagnostic potential of sphingolipids specific signature in EV in STEMI patients versus matched control subjects.

## Results

### Study cohort clinical profile

We analyzed peripheral venous blood samples of patients with a diagnosis of STEMI (n = 7) on presentation to the emergency department before primary percutaneous coronary intervention (PCI), as well as 24 h (hours) post-intervention, as compared to controls (n = 9), matched for cardiovascular risk and demographic parameters. The median time from chest pain onset to the presentation at the emergency department was 3 h. PCI was performed within 1 h from the first medical contact for all STEMI patients. Mean age was 62 ± 10.6 years, 68.8% were males, with a high prevalence of hypertension, and dyslipidemia. Study groups did not significantly differ from one another with respect to systolic and diastolic pressure, BMI, white blood cells count, creatinine, glomerular filtration rate, glycemia, total cholesterol, HDL, and triglycerides. As expected, patients with a diagnosis of STEMI displayed higher levels of high-sensitive (hs)-troponin and C-reactive protein (CRP), and lower left ventricular ejection fraction (LVEF%) at echocardiography 24 h post-PCI as compared to controls (*p* < 0.05 for all comparisons). Clinical and biochemical parameters are reported in Table 1.

**Table 1:** Patient profile.

Variable	Ctrl [n = 9]	STEMI [n = 7]	*P-*value
Age (years)	59 ± 8.3	65 ± 13.1	0.357
Sex (ref. male)	5 (55.6)	6 (85.7)	0.197
Hypertension (ref. yes)	5 (55.6)	4 (57.1)	0.949
Dyslipidemia (ref. yes)	4 (44.4)	5 (71.4)	0.280
Systolic blood pressure (mmHg)	128 ± 7.9	126 ± 27.6	0.815
Diastolic blood pressure (mmHg)	82 ± 5.1	74 ± 12.2	0.124
BMI (Kg/m^2^)	27.7 ± 2.42	28.5 ± 5.23	0.666
hs-troponin (ng/L)	8 ± 12.8	322 ± 368.8	**0.022**
WBC (n/uL)	7202 ± 2216.2	9729 ± 3096.1	0.077
Creatinine (mg/dL)	0.77 ± 0.13	0.96 ± 0.24	0.084
GFR (mL/min)	96 ± 7.6	90 ± 35.4	0.694
CRP (mg/L)	2.1 ± 0.98	7.3 ± 10.8	**0.023**
Glycemia (mmol/L)	5.3 ± 0.63	7.0 ± 1.32	0.072
Total cholesterol (mmol/L)	5.2 ± 1.16	4.9 ± 1.40	0.604
HDL (mmol/L)	1.8 ± 1.03	1.1 ± 0.19	0.079
Triglycerides (mmol/L)	1.3 ± 0.94	1.5 ± 1.40	0.712
LVEF at echo (%)	62 ± 2.5	53 ± 6.4	**0.007**

Clinical and biochemical characteristics of patients diagnosed with ST-segment elevation myocardial infarction (STEMI) compared to controls (Ctrl). High-sensitive (hs)-troponin, WBC (White Blood Cells), creatinine, GFR (Glomerular Filtration Rate), CRP (C-Reactive Protein), glycemia, total cholesterol, HDL, and triglycerides were assessed at presentation to the emergency department for STEMI patients. LVEF (Left Ventricular Ejection Fraction at echocardiography) was assessed 24 h after reperfusion. Data are expressed as mean ± SD, or absolute number (%) when appropriate. *P-*value < 0.05 was considered significant and indicated by bold characters.

### EV isolation and characterization

**Figure 1:**
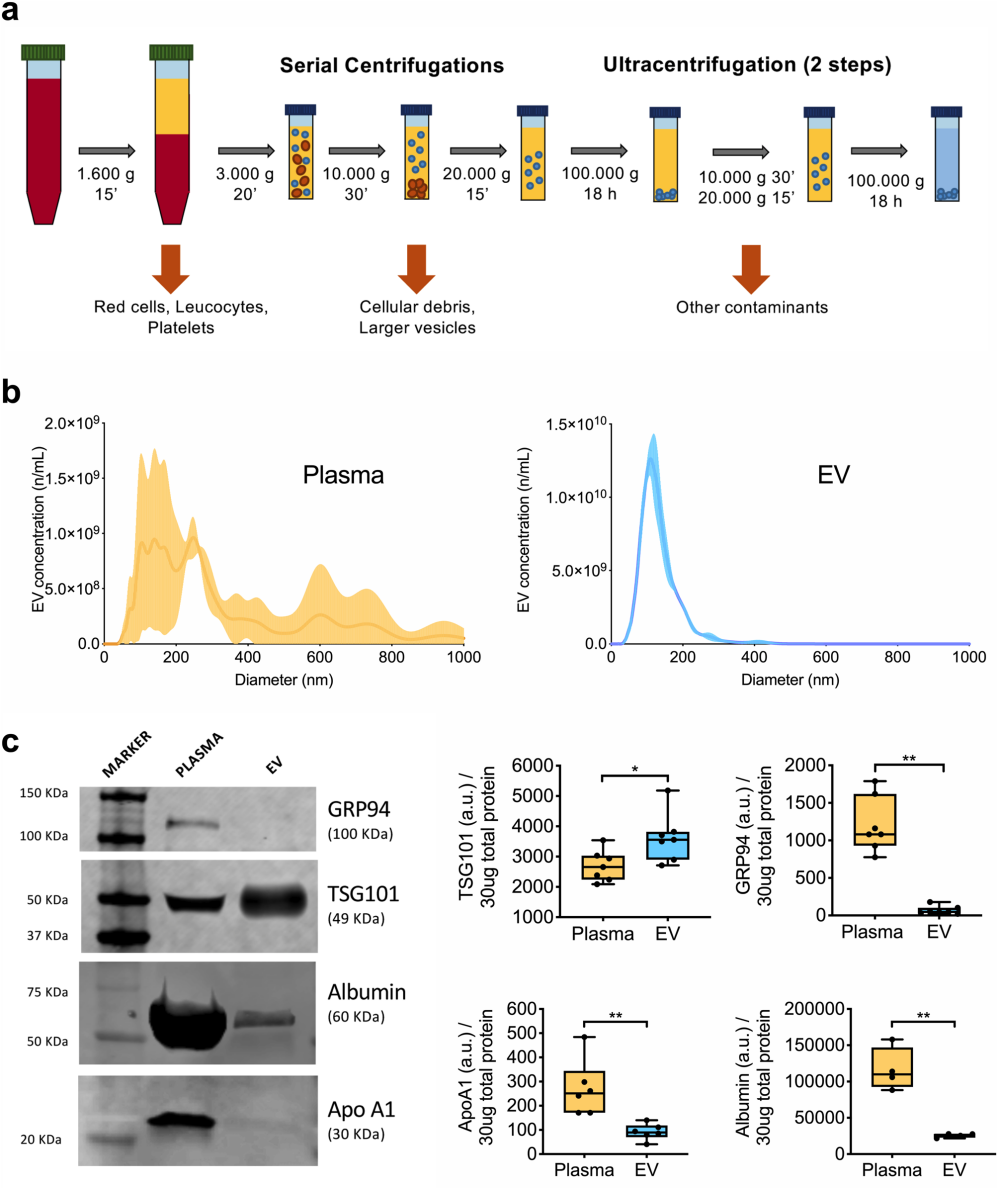
EV isolation and purification from plasma sample. (**a**) Schematic of EV isolating protocol; after serial centrifugations step to remove intact cells, cellular debris and large particles, free-platelet plasma underwent two-step ultracentrifugation to precipitate extracellular vesicles. (**b**) Representative cumulative distribution plot at nanoparticle tracking analysis for plasma (yellow curve) and EV (blue curve). (**c**) Western blot analysis for TSG101, as EV specific intraluminal marker, and potential contaminants (GRP94, apolipoprotein A1, and albumin) in representative patients (n = 7), as compared to whole plasma. Level of expression are reported as arbitrary unit (a.u.) in 30 μg of total protein EV lysate. A representative immunoblot is shown referring to STEMI patient at pre-PCI evaluation. Uncropped membranes are shown in Fig. S1a,b.

The classical EV isolation protocol using a serial centrifugation steps, here has been implemented to further remove cellular debris and blood contaminants. EV were isolated from 200 μL of platelet-free plasma by double series of sequential centrifugations as depicted in Fig. 1a. The final solution enriched in extracellular vesicles is referred hereby as EV.

Nanoparticle tracking analysis (NTA) was performed in parallel in pre-cleared plasma and enriched EV, the latter showed typical dimensional profile of small EV (90% of total particles were less or equal 227 ± 38.5 nm), with no contamination of larger particles (Fig. 1b). Western blot analysis confirmed enrichment of small EV also referred elsewhere as exosomes^20^, based on the TSG101 expression (1.3-fold increase; *p* = 0.013), with negligible presence of antigens gauging the presence of cellular debris and/or lipoparticles as compared to plasma (Fig. 1c and Figs. S1a,b, S2). As expected, whole plasma was positive for albumin, apolipoprotein APOA1, and for the endoplasmic reticulum chaperone marker GRP94^31^. Albumin and ApoA1 were also present in EV preparations, although their expression was markedly reduced (4.1- and 2.8-fold decrease, respectively; *p* < 0.01), whereas GRP94 was hardly expressed (22.6-fold decrease; *p* < 0.001; Figs. 1c, S1a,b, S2). Collectively, these data indicate that the plasma EV preparations obtained by our optimized protocol were enriched in exosomes with minimal presence of contamination in terms of albumin and lipoproteins, and negligible presence of cellular debris.

**Figure 2:**
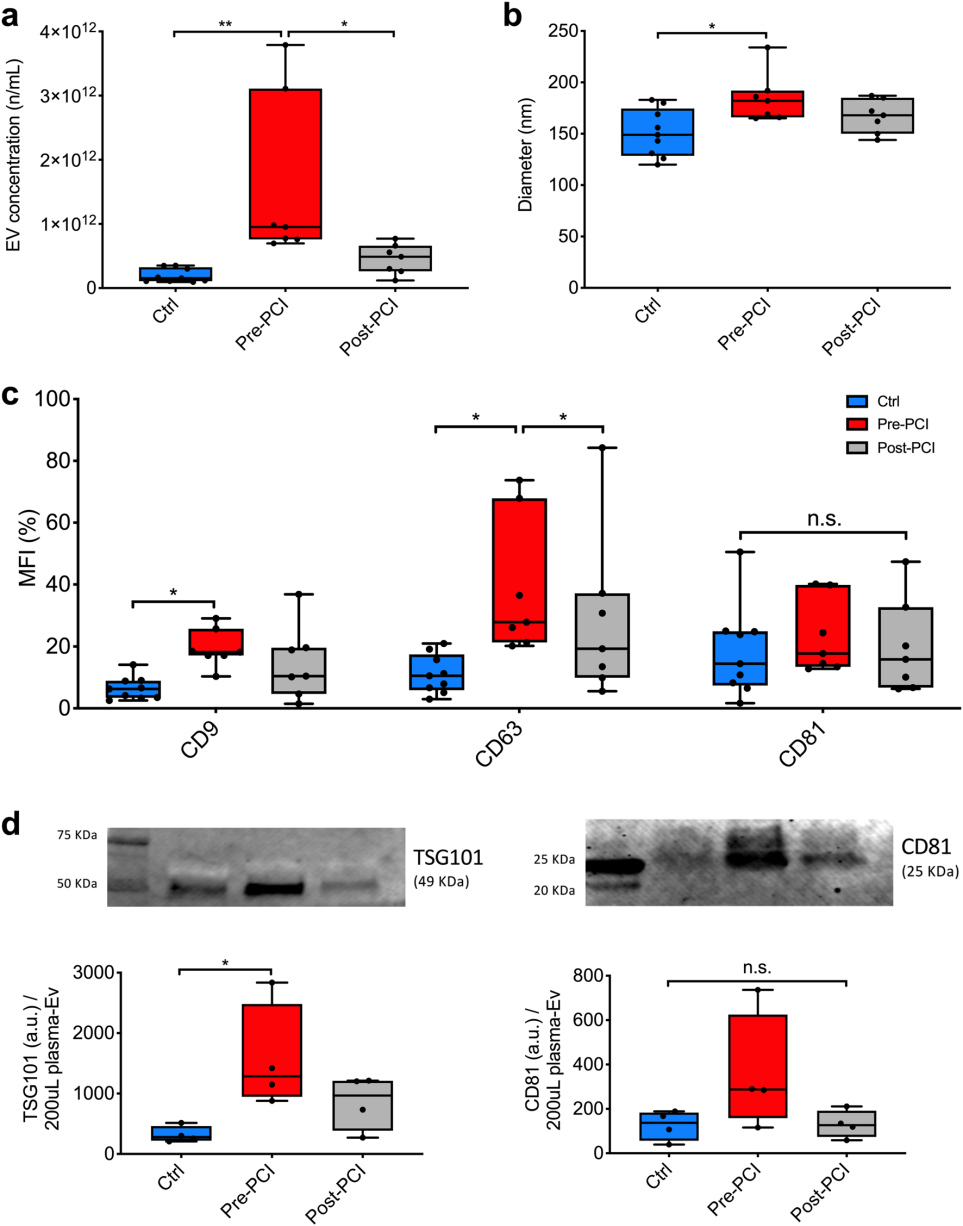
EV characterization. (**a**, **b**) EV concentration (particles/mL) and nanometer diameter (nm) were assessed by NTA in patients with a diagnosis of STEMI (n = 7) before PCI and after 24 h from reperfusion, as compared to controls (Ctrl; n = 9). (**c**) Percentage value of median fluorescence intensity (% MFI) for CD9, CD63, and CD81 assessed by flow cytometry. (**d**) Western blot analysis for EV specific markers (TSG101 and CD81) in representative patients (n = 4). Level of expression are reported as arbitrary unit (a.u.) in patients with STEMI before and 24 h after reperfusion, as compared to controls. Uncropped representative membranes are shown in Fig. S1c,d. Data and statistical analysis are reported in Table S1. *p < 0.05; **p < 0.01.

Once the reliability of the isolation protocol had been assessed, we sought to evaluate differences between patient and matched controls in terms of EV-plasma concentration. NTA analysis clearly highlights a significant increase of plasma EV concentration in patients with STEMI as compared to controls (9.5E11 particles/mL [7.6E11–3.1E12] vs*.* 1.6E11 particles/mL [1.1E11–3.3E11]; *p* = 0.003). After reperfusion, the amount of circulating EV significantly decreased (4.9E11 n/mL [2.6E11–6.6E11]; *p* = 0.036; Fig. 2a). Moreover, EV size as measured by diameter was higher in patients with STEMI at pre-PCI evaluation compared to controls (182 nm [166–192] vs*.* 149 nm [129–175] *p* = 0.033; Fig. 2b and Table S1).

To further exclude the presence of cross-contaminating particle with similar size, plasma EV were then analyzed based on the expression of tetraspanins (CD9, CD63, CD81) by flow cytometry. To overcome the issue of size-related limit of detection, EV were bound to capture beads coated with specific antibodies. Mean fluorescence intensity values for CD9 and CD63 were significantly increased in patients with STEMI compared to controls (18.2 [17.1–25.7] versus (vs.) 8.8 [4.9–14.9] and 27.7 [21.3–67.9] vs. 13.4 [8.6–29.0]; *p* < 0.05 for both comparisons), and an increasing trend was observed also for CD81 (Fig. 2c). Levels of tetraspanin antigens decreased after reperfusion (Table S1). To validate our findings in accordance to International Society of Extracellular Vesicles (ISEV) recommendation^32^, we performed a semiquantitative analysis by WB for both surface and luminal EV specific proteins in a subset of subjects (4 controls vs. 4 patients with STEMI before and after PCI). Immunoblot assays showed an increase of canonical EV markers such as TSG101 and CD81 in pre-PCI samples as compared to control ones (1284.2 [946.4–2482.0] vs*.* 280.2 [220.7–561.5] arbitrary unit -a.u.- and 287.1 [158.1–624.8] vs. 137.2 [56.2–183.5] a.u., respectively) (Figs. 2d and S1c,d). Finally, to explore the putative origin of circulating EV, we profiled their surface by using multiplex assay to simultaneously analyze 37 antigens (Table S2 and Fig. S3). Tetraspanins (CD63, CD9 and CD81), specific markers of EV^32^, were highly expressed on the surface of vesicles. Moreover, EV were found positive for markers of activated platelets (CD62p, CD41b, and CD42a), endothelium (CD31), and leukocytes (CD40). Markers of inflammatory cells (e.g. CD2, CD3, CD4, CD8, CD14, CD19, CD20, CD45; Table S2 and Fig. S3) were also found in a minor proportion of EV (less than 5%).

### Sphingolipids composition of EV after myocardial infarction

**Figure 3:**
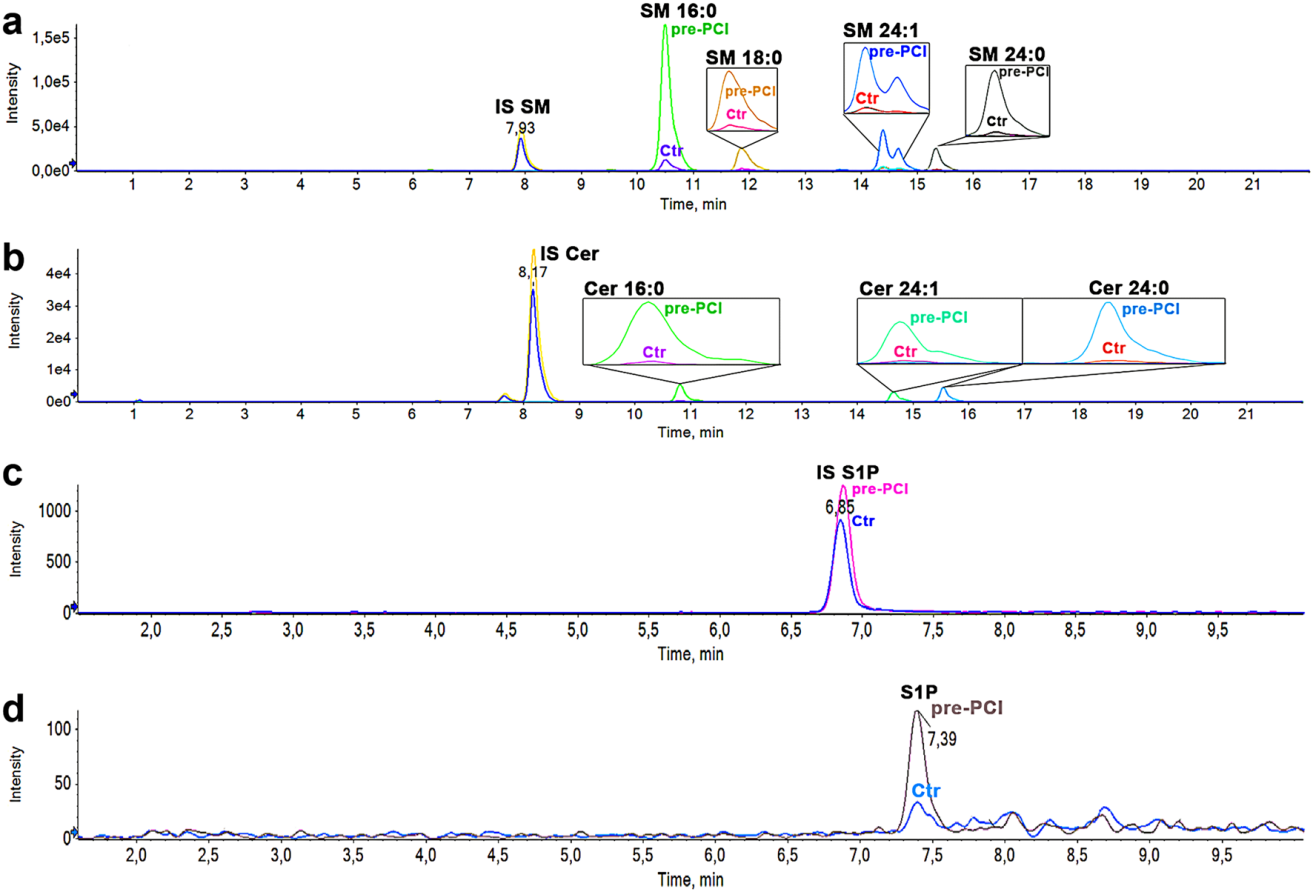
Sphingolipid chromatographic traces by LC–MS/MS. Overlay of the chromatographic traces of EV samples (STEMI at pre-PCI evaluation *vs.* Ctrl). (**a**) Analysis of sphingomyelins, (**b**) ceramides and dihydroceramides, (**c**, **d**) IS S1P and S1P. Different chemical species are indicated in bold (IS, Internal Standard used in each analysis to counterbalance experimental variation; Tables S3, S4). Split peak of SM 24:1 (panel **a**) is due to the double bond positional isomerization in the fatty acid. Panels (**a**, **b**) refer to the same analysis for sphingolipid determination (Column ACQUITY UPLC BEH C8 1.7 μm 2.1 × 100 mm). Panels (**c**, **d**) refer to a different analysis on the same sample, with conditions optimized for sphingosine bases (Column Restek Raptor C18 2.7um 2.1 × 100 mm).

Sphingolipids composition was evaluated by LC–MS/MS analysis in whole plasma over isolated EV (Tables S3–S6). Representative chromatographic traces for EV samples (STEMI patient at pre-PCI evaluation vs. control patient) are shown in Fig. 3.

**Figure 4:**
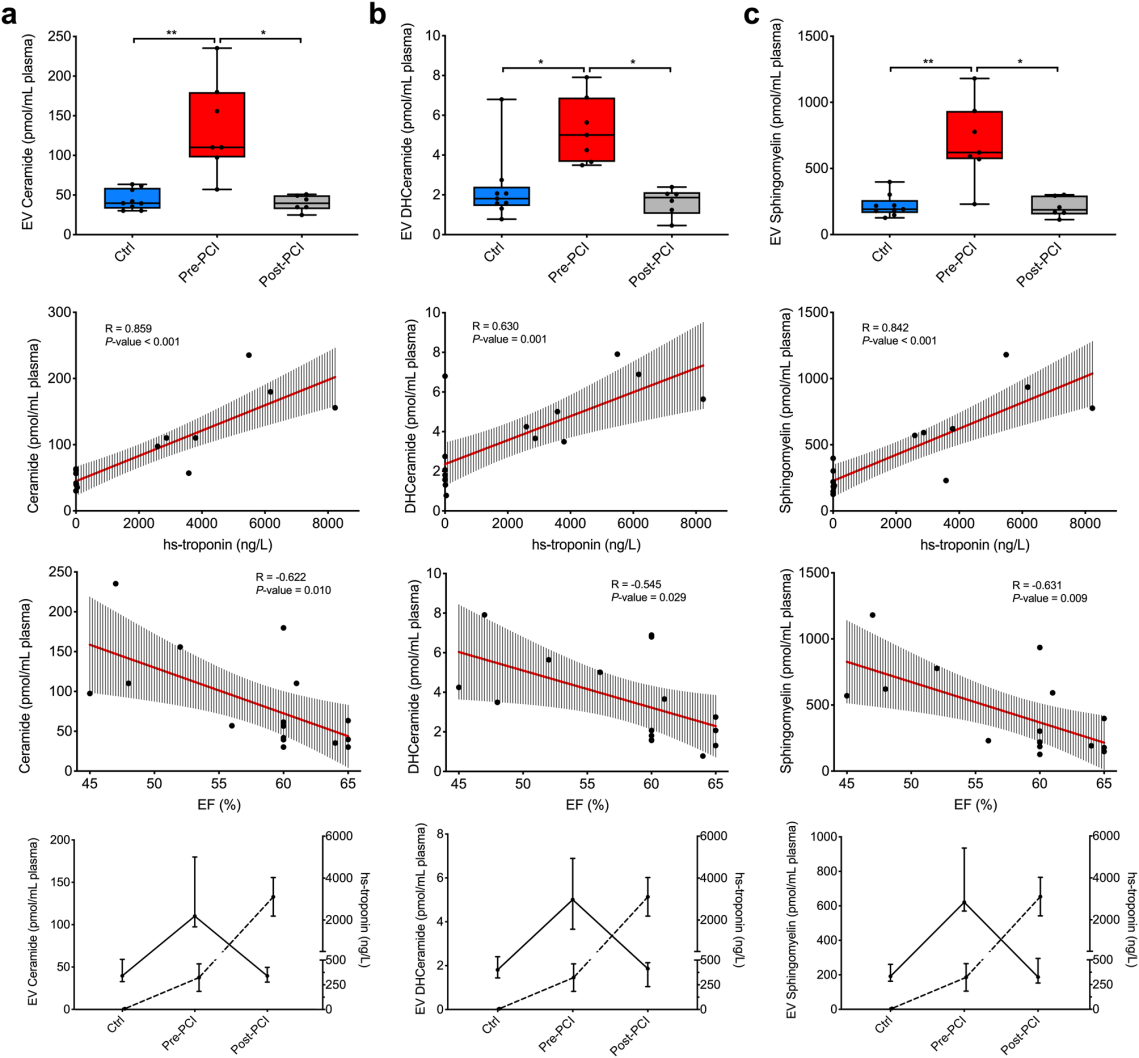
EV Sphingolipid composition following MI. Ceramide, dihydroceramide, and sphingomyelin EV content in patients with STEMI diagnosis (n = 7) before PCI and after 24 h from reperfusion, versus healthy controls (Ctrl; n = 9; first row). Correlations are shown for total ceramides, dihydroceramides, and sphingomyelins to high-sensitive (hs)-troponin peak (ng/L; second row) and left ventricular ejection fraction at echocardiography at 24 h after reperfusion (LVEF; third row). Regression lines and 95% confidence intervals are shown. Curves for sphingolipids levels and hs-troponin in controls and STEMI patients pre- and post-PCI are reported in the last row. Data and statistical analysis are reported in Tables S5–S7. *p < 0.05; **p < 0.01.

EV total content of ceramides, dihydroceramides, and sphingomyelins significantly increased in patients with STEMI, as compared to controls (110.1 pmol/mL [97.4–179.9] vs. 39.7 pmol/mL [32.8–59.1], 5.0 pmol/mL [3.7–6.9] vs. 1.8 pmol/mL [1.4–2.4], and 621.0 pmol/mL [570.2–934.9] vs. 191.6 pmol/mL [162.9–261.2]; *p* < 0.05 for all comparisons; Fig. 4 and Table S5). The sphingolipids concentration in EV was directly correlated to the peak level of high sensitive troponin (hs-troponin) reached by each patient (R ranging between 0.630 and 0.859; *p* < 0.01 for all comparisons) and inversely correlated to ejection fraction measurements via ultrasound echocardiography at 24 h post reperfusion (R ranging between − 0.545 and − 0.631; p < 0.05 for all comparisons; Fig. 4 and Table S7). Finally, EV sphingolipids concentration decrease 24 h after reperfusion (*p* < 0.05). As a result, the peak in EV sphingolipid concentration anticipated the peak of hs-troponin, which was reached at or after 24 h from PCI (Fig. 4).

The presence of ceramides, dihydroceramides, and sphingomyelins in plasma was 500 to 700-fold higher as compared to EV. Similarly, to what found in EV, plasma sphingomyelin concentration significantly increased after STEMI before PCI and fell back to control level at 24 h after reperfusion (pre-PCI 420.6 pmol/mL [399.7–461.5; controls 169.3 pmol/mL [154.4–184.9]; post-PCI 149.9 pmol/mL [129.8–176.2]; *p* < 0.001). Moreover, sphingomyelin plasma levels significantly correlated with both hs-troponin and ejection fraction (Fig. S4 and Tables S6, S7). On the contrary, plasma levels of ceramides and dihydroceramides did not differ between STEMI patients and controls; likewise, neither hs-troponin, nor ejection fraction significantly correlated with their concentrations in plasma (*p* > 0.05 for all comparisons; (Fig. S4 and Tables S6, S7). In addition, ceramide, dihydroceramide, and sphingomyelin total content within isolated EV was directly correlated to circulating white blood cells (R 0.591, 0.504, and 0.578; *p* < 0.05), contrary of plasma sphingolipids (Table S7).

**Figure 5:**
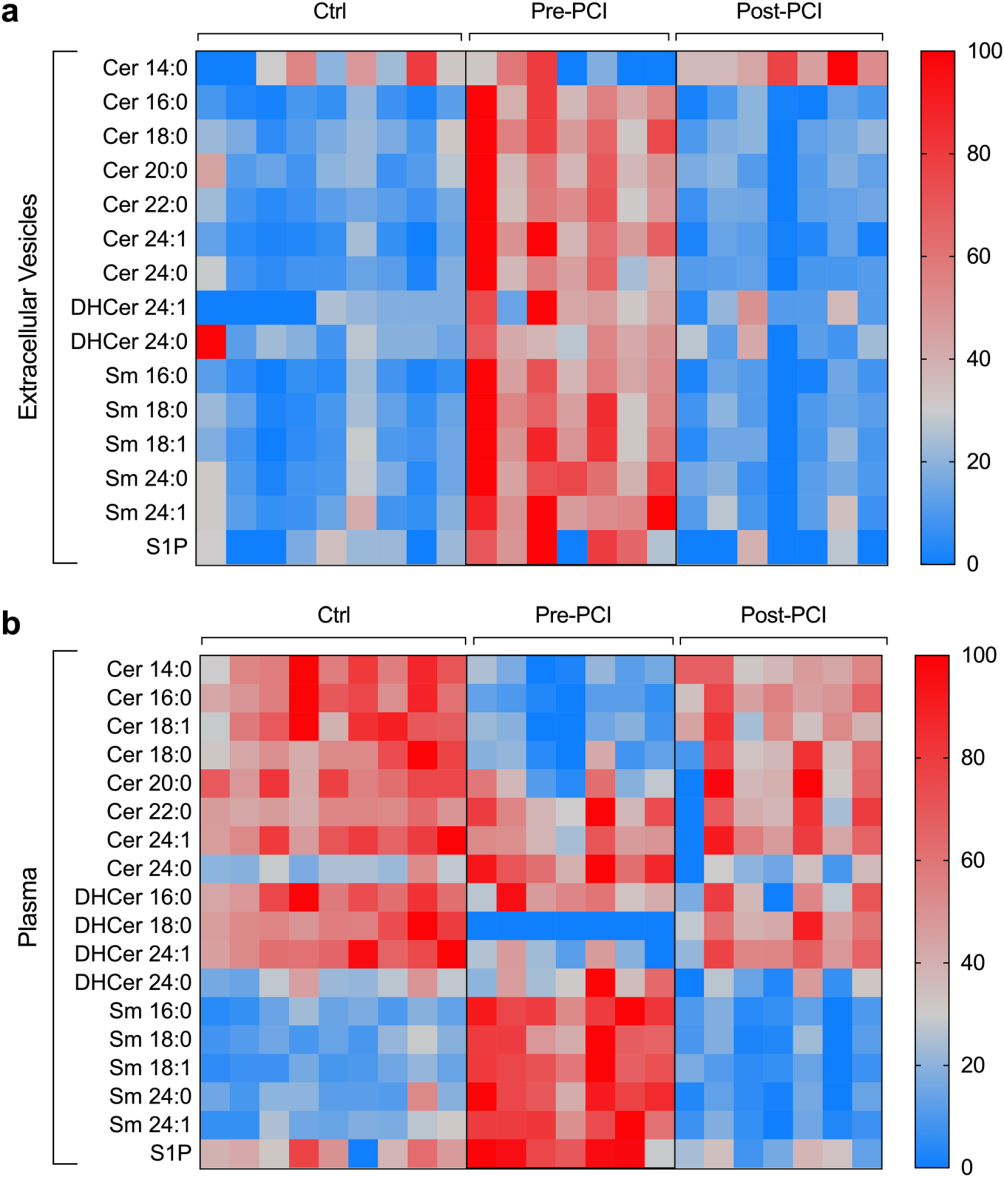
EV Sphingolipid composition compared to whole plasma. Heat map of specific EV composition ceramides, dihydroceramides, and sphingomyelins in extracellular vesicles (**a**) as compared to plasma (**b**), in patients with STEMI (n = 7) before PCI and after 24 h from reperfusion, as compared to controls (Ctrl; n = 9). Low/high sphingolipid levels are represented in blue/red color. Data and statistical analysis are reported in Tables S5 and S6.

We then investigated the contribution of 20 different species of sphingolipids to the total amount of differential expressed lipids in whole plasma versus isolated EV (Fig. 5). LC–MS/MS analysis showed that ceramide 18:1, and dihydroceramides 16:0 and 18:0 were not detectable in EV, whereas dihydroceramide 18:1 was not detectable in EV as well as in plasma. All the other ceramide, dihydroceramide, and sphingomyelin species except for ceramide 14:0 significantly increased after STEMI in EV (*p* < 0.05 for all comparisons; Table S5), with ceramide 16:0 being the most represented among ceramides, and sphingomyelin 16:0 among sphingomyelins. On the contrary, all sphingomyelin species and ceramide 24:0 increased after myocardial ischemia in plasma, whereas ceramides 14:0, 16:0, 18:1, 18:0, and 20:0 and dihydroceramides 18:0 and 24:0 significantly decreased in patients with a diagnosis of STEMI as compared to controls (Table S6).

**Figure 6:**
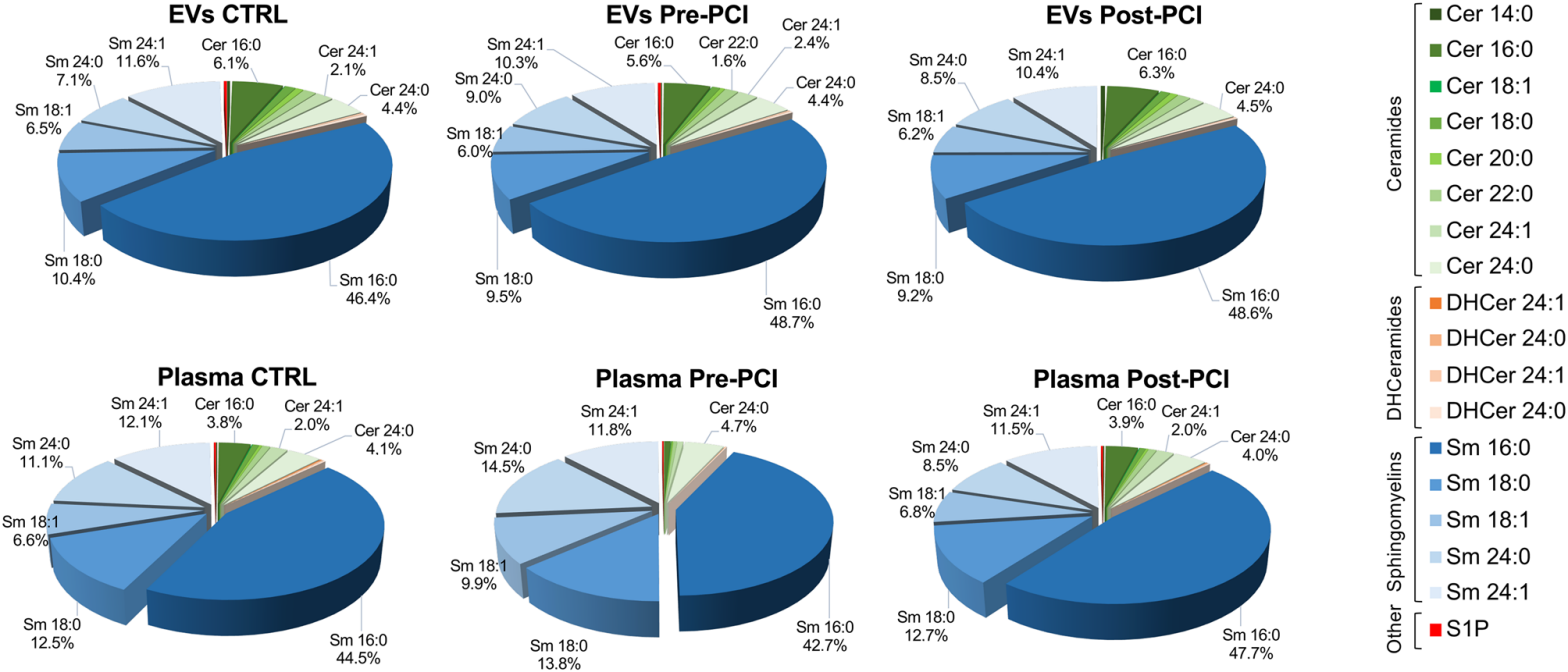
Sphingolipid composition in EV and plasma. Sphingolipid composition (percentage values) in EV versus plasma, in patients with a diagnosis of STEMI (n = 7) before PCI and after 24 h from reperfusion, as compared to controls (Ctrl; n = 9). *Cer* Ceramides, *DHCer* Dihydroceramides, *Sm* Sphingomyelins, *S1P* Sphingosine-1-phosphate. Data analysis: see Table S8.

Among ceramide metabolites, EV levels of sphingosine-1-phosphate were increased in patients with STEMI as compared to controls (2.58 pmol/mL [1.05–3.13] vs. 0.86 pmol/mL [0.0–1.0]) and decreased after 24 h reperfusion (0.0 pmol/mL [0.0–1.1]; *p* = 0.022). The same trend was observed in plasma, where sphingosine-1-phosphate levels increased after STEMI (*p* = 0.003; Tables S5, S6). Overall, sphingomyelins were the most represented species both in EVs and whole plasma, independently from the diagnosis (82.0–83.4% and 86.8–92.7% of sphingolipid content, respectively in EVs and in plasma) (Fig. 6).

To exclude confounding effects due to the presence of contaminants, we verified the presence of correlations between albumin or ApoA1 and sphingolipids levels in EV (Table S9 and Fig. S5). We found no significant correlations with sphingolipid levels. Furthermore, patients were stratified for fasting conditions (Table S10) so that levels of lipids were re-analyzed in STEMI patients at pre-PCI stage according to this stratification. We found no difference between patients with STEMI in fasting conditions as compared to those sampled at not fasting conditions (p > 0.05 for all comparisons).

### Diagnostic modelling

**Figure 7:**
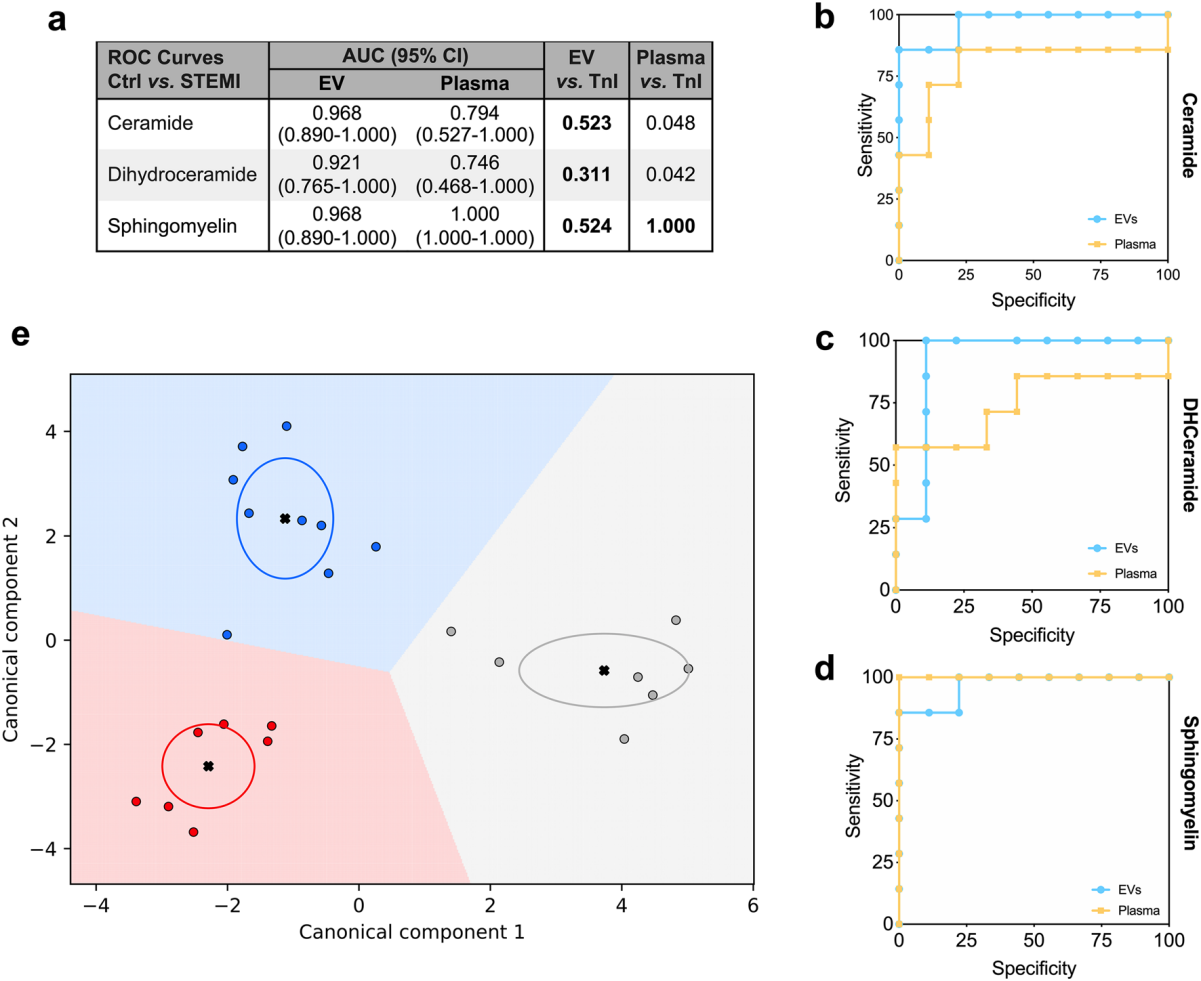
Sphingolipid signature after myocardial infarction. (**a**) The confusion matrix shows the area under the curve (AUCs) after ROC curve analysis for ceramides, dihydroceramides, and sphingomyelins in plasma and plasma derived EV. AUCs were compared to hs-troponin I (TnI), as referral for the diagnosis of STEMI. (**b**–**d**) ROC curves for ceramides, dihydroceramides, and sphingomyelins content in plasma (yellow line) *vs.* EV (blue line). (**e**) Canonical plots representing patient distribution according to diagnosis and linear weighted combination of ceramide, dihydroceramide, and sphingomyelin levels in EV. Red, grey, and blue points represent subjects with STEMI before PCI, 24 h after reperfusion, and controls, respectively. Crosses indicate mean values of (canonical-1; canonical-2) for each category. Ellipses include patients with a linear combination coefficient that falls within the mean ± SD.

To verify the diagnostic relevance of the three main classes of lipids detected in EV over those enriched within whole plasma, ROC analysis was performed, and AUC was calculated. The gold standard biomarker for STEMI, hs-troponin was used as reference. Except for sphingomyelin, AUC of EV lipids was higher as compared to whole plasma. AUCs for EV-derived ceramides and dihydroceramides, as well as sphingomyelins from both EV and plasma, were not different from hs-troponin (*p* > 0.005; Fig. 7a–d). Moreover, EV levels of ceramides, dihydroceramides, and sphingomyelins were combined in a specific signature using supervised machine learning algorithms via linear discriminant analysis. Canonical plot showed that this approach correctly discriminated all the patients with a diagnosis of STEMI (before and after primary PCI), from controls (Fig. 7e). Collectively, these data suggest the relevance of EV lipid signature as biomarker compared to hs-troponin for the diagnosis of STEMI.

## Discussion

This study addresses sphingolipid profiling of circulating EV in patients with a diagnosis of STEMI, before and 24-h after primary PCI against control subjects as promising theranostic tool. Specifically, here we applied lipidomics with supervised machine learning algorithms to pinpoint EV sphingolipid signature in order to accurately discriminate patients with STEMI before and after PCI and controls. Our group recently described that in a preclinical rat model of MI, the large majority of sphingolipids species are increased in EV derived from plasma^27^. Here, we further confirmed modulation of lipid EV membrane in cardiovascular patients as well.

Ceramide, dihydroceramide, and sphingomyelin EV content was significantly increased in patients with STEMI at pre-PCI evaluation, and correlated to the highest hs-troponin reached by each patient and to the ejection fraction at echocardiography 24 h post-PCI, as indices of myocardial injury severity. The levels of three lipid species in EV were not inferior to troponin as potential diagnostic biomarker for acute myocardial infarction. Regarding whole plasma composition, as for ceramides and dihydroceramides, the differences between patient and controls subjects revealed a trend in their decrease.

Sphingolipids are one of the major lipid constituents of eukaryotic cells and, beside their structural role, they also function as signaling molecules for maintenance of cell membrane integrity, apoptosis, cellular damage or oxidative stress response, cell growth, senescence and migration, endothelial cell function, and inflammatory signaling^13,33–35^. Thus, profiling of circulating sphingolipids in patients may offer relevant information and allow identification of specific disorders and disrupted metabolic pathways^35^. Such diagnostic approach can be further implemented by recent technological advances in mass spectrometry (MS), which allows precise characterization of different lipid molecules^2^. Here, we provided additional sensitivity by quantifying sphingolipid species of circulating EV. In STEMI patients, systemic EV are mainly composed of vesicles that are released in the blood as result of acute thrombosis and inflammation^36^. Accordingly, we observed the expression of surface antigens from activated platelets (CD62p, CD41b, and CD42a), endothelium (CD31), and leukocytes (CD40), as previously described^36^. A minor proportion was positive for markers of inflammatory cells (e.g. CD2, CD3, CD4, CD8, CD14, CD19, CD20, CD45). A crucial aspect in characterizing circulating EV is represented by the cross-contamination with lipidic particles. To overcome this issue and to minimize the presence of contaminants without affecting the yield of EV enabling consistent MS analysis, we applied a double series of centrifugation steps interspaced by two washing steps. This allowed us to isolate plasma EV with reasonable specificity as demonstrated by western blotting analysis, in line with previous study showing that UC method still represent a suitable method for isolating EV from plasma^26^. Consistently, apolipoprotein A1 and albumin were not correlated to sphingolipid levels, and fasting conditions did not modify lipid profile in patients with STEMI.

Healthy individuals usually present a concentration of circulating plasma EV ranging between 10^7^ and 10^9^ EV/mL^37^, whereas lipoproteins are about 10^16^ particles/mL^22^. Concomitantly, we found that sphingolipids were significantly more abundant in plasma than in EV (Tables S5 and S6). However, the significant increase in sphingolipids observed in EV of STEMI patients at pre-PCI stage, was mirrored in plasma by sphingomyelins (C16 to C24), and by ceramide C24:0 species. In STEMI patients at pre-PCI evaluation, EV sphingomyelin species (C16 to C24) and ceramide 24:0 were increased, as compared to controls, whereas they decreased after reperfusion. This is consistent with previous studies demonstrating an increase of circulating sphingomyelins 16:0 and 24:0 in patients with atherothrombotic diseases, such as MI or ischemic stroke^5,38^. Indeed, some sphingolipids species are enriched in atherosclerotic plaques^39^, thus possibly suggesting a role in their pathogenesis. Similarly, ceramides 16:0, and sphingomyelin have been previously associated with negative cardiovascular outcome and worsening of cardiac function in heart failure^1^. Moreover, the plasma signature of specific ceramides and dihydroceramide species has been proven as prognostic reference for major adverse cardiovascular events in patients with acute CAD^7,8,40^.

In our study we further confirmed such findings for plasma levels of sphingomyelin, but neither for ceramide (C14 to C22) nor dihydroceramide (C16 to C24). Although the concentration sphingolipid species in control group is in line with the reference range of circulating sphingolipid in human plasma from healthy subjects^41^, our study suffers of limited numerosity of included patients, which can partially explain this discrepancy. Moreover study design and blood sampling modality (different anticoagulants) may influence LC–MS/MS results^41,42^.

Notably, the increase of different ceramide species has been shown to concur to the upregulation of different acute phase proteins, and to leucocytes recruitment and activation^43–45^. Moreover, it is widely acknowledged that S1P possesses pleiotropic functions and orchestrates many cellular processes involved in cardiovascular pathophysiology^46^. This is consistent with our findings showing a direct correlation between sphingolipid EV content and leucocyte count. Inflammatory environment may also partially explain the increase in overall sphingolipids species carried by EV, as the secretion of inflammatory cytokines (interferon-γ, TNF-α, and IL1β) together with cellular stress, stimulate the de-novo synthesis of ceramides through the enzymatic activity of the dihydroceramide desaturase, which catalyze the conversion of dihydroceramides in ceramide^13,47,48^.

Sphingolipids and EV are not only measurable indicator of some biological state or condition in cardiovascular disease, but also causative agents in CAD progression. Growing evidence suggests that the blood levels of ceramides are associated with the exacerbation of myocardial ischemic disease and its complications^7,40,49^. Studies on murine models revealed that pharmacological inhibition of the synthesis of ceramides prevents heart failure after myocardial ischemia, decreasing ventricular remodeling, fibrosis, and inflammatory infiltrate^8,43,50^. Similarly, the administration of an inhibitor of neutral SMases which hydrolyzes sphingomyelin to ceramide, thus impairing EV biogenesis, namely GW4869, reduced the increase of circulating inflammatory EV after MI in rats, resulting in the preservation of left ventricular ejection fraction^27^.

To the best of our knowledge, this is the first study combining a protocol to isolate EV that meets current recommendation in terms of purity^32,51^, a robust targeted lipidomic MS-based platform for the accurate evaluation of less-abundant lipid species, and a machine learning algorithm to model a specific sphingolipid signature in STEMI patients. In such prospective, we demonstrated that EV sphingolipids can provide a theranostic platform to gain new information about disease status. This represents critical information to infer insights into developing novel biomarkers. This is further strengthened by considering that such approach is significantly different from traditional cardiovascular diseases lipid biomarkers such as cholesterol^6^.

Nonetheless, limitations should be acknowledged as well. First of all, limited number of patients enrolled in the study, which will have to be replicated in larger cohorts. Plasma is a complex biofluid and we acknowledged residual albumin contamination in EV samples, which might affect EV count by NTA, and therefore should be taken in consideration; nevertheless, circulating EV increase in STEMI patients has been largely documented^52,53^. Moreover, antithrombotic treatment administered to patients with MI could have affected sphingolipid composition. However, all STEMI patients received standard treatment (i.e. dual anti-platelets therapy and unfractioned heparin); hence, we can assume that the comparison between STEMI patients at pre-PCI vs*.* 24 h post-PCI could not have been biased. Finally, the use of ultracentrifugation is not clinically feasible; therefore alternative, yet highly efficient, isolating method should be optimized to be translated into clinical practice.

In conclusion, this is the first description of a dynamic change in EV sphingolipid signature after STEMI in humans, thus attracting a considerable interest in the context of personalized medicine for patients with CAD. In such perspective, EV sphingolipid profiling before and after reperfusion could be used as prognostic tool for STEMI patient. In such scenario, circulating EV modulation may be used as therapeutic strategy to improve their prognosis.

## Methods

### Patient selection and blood sampling

Subjects were enrolled at the Fondazione Cardiocentro Ticino (Lugano, Switzerland). The study protocol complied with Helsinki Declaration and was approved by the “Ticino Cantonal Ethical Committee”, Switzerland, and informed written consent was obtained from each participant. Patients were diagnosed with STEMI according to the European Society of Cardiology guideline^29^; peripheral blood samples were collected on the presentation to the emergency department before primary PCI and after 24 h from reperfusion (named as pre-PCI and post-PCI samples). Patients were excluded in case of: (1) Age > 85 years; (2) Chest pain onset > 6 h; (3) Glomerular filtration rate < 30 mL/min; (4) Atrial fibrillation, ventricular tachycardia, ventricular fibrillation, cardiac arrest, or cardiogenic shock with indication to invasive device assistance; (5) Other non-ischemic cardiac disease (severe heart valve disease, chronic heart failure), or acute/chronic inflammatory disease (e.g. auto-immune disease, cancer, infections). Asymptomatic subjects underwent computed tomography or magnetic resonance imaging which documented the absence of significant coronary stenoses (> 30% diameter reduction) and were recruited as controls. All patients received a standard treatment with dual anti-thrombotic therapy (aspirin 150–300 mg plus either clopidogrel 300–600 mg, or prasugrel 60 mg, or ticagrelor 180 mg) and unfractioned heparin 70–100 IU/Kg. Control subjects, STEMI patients at post-PCI evaluation and 4 of 7 STEMI patients at pre-PCI evaluation were sampled in fasting conditions.

### EV isolation

Blood was collected in sodium citrate tubes and immediately centrifuged at 1600×*g* for 15 min at 4 °C to avoid platelet activation and separate plasma from cellular components. All hemolytic plasma samples were excluded from the study. Free-platelet plasma was differentially centrifuged at 3000×*g* for 20 min to remove cellular debris, and then at 10,000×*g* for 30 min, and 20,000×*g* for 15 min to remove apoptotic bodies and large particles^27^. EV were obtained from 300uL of free-platelet plasma by ultracentrifugation at 100,000×*g* (18 h) using a Beckman Optima Max-TL ultracentrifuge (Beckman Coulter). Plasma EV enriched pellet were resuspended in PBS 1X and underwent washing steps by further centrifuge steps at 10,000×*g* for 30 min, and 20,000×*g* for 15 min, with a final step of ultracentrifuge at 100,000×*g* (18 h) (Fig. 1a) as previously described^27^. Plasma-EV pellets were resuspended in 100 μL PBS, pH 7.4, and then stored at − 80 °C, prior to analysis.

### EV characterization

EV concentration and size were measured by nanoparticle tracking analysis (NTA), using NanoSight LM10 (Malvern Instruments, United Kingdom) equipped with a 405 nm laser and NTA 2.3 analytic software. Free-platelet plasma and EV samples were diluted 1:2000 in PBS 1X and exposed to a laser light source. Brownian movements of EV were recorded by a camera and nanoparticles size and number per mL were calculated by Stokes–Einstein equation. Three videos of 60 s were recorded for each sample to perform the analyses. Nanoparticles concentration was reported as number of particles per mL (n/mL).

After ultracentrifugation, purified EV were analysed by MACSPlex human Exosome Kit (Miltenyi, Bergisch Gladbach, Germany) with MACSQuant Analyzer-10 flow cytometer (Miltenyi, Bergisch Gladbach, Germany), as previously described^36,54^. EV samples were incubated overnight with capture-beads coated with specific antibodies for 37 EV surface antigens, and then with detection antibodies against CD9, CD63, CD81. The median fluorescence intensity (MFI) was analyzed after normalization for blank control.

Free-platelet plasma and EV total proteins were extracted with RIPA buffer (25 mM Tris pH:7,4; 150 mM NaCl; 1 mM EDTA; 1% Igepal CA630; 1% Na-deoxycholate; 1.01% sodium dodecyl sulphate; SDS) supplemented with protease inhibitors (SIGMAFAST Protease Inhibitor Tablets, Sigma) for 30 min at 4 °C under agitation. Protein concentrations were determined using BCA kit (Sigma). Equal amount of total proteins (30 μg) or equal volume of sample were boiled with Laemmli SDS sample buffer 6X (0.375 M Tris–HCl pH 6.8, 12% SDS, 60% glycerol, 0.6 M DTT, 20%(v/v) b-mercaptoethanol, 0.2% (w/v) bromophenol blue; VWR International), separated on 4–20% Mini-PROTEAN TGX Precast Gel (Bio-Rad), and transferred onto PVDF membranes with a semi-dry transfer system (Bio-Rad)^27^. The membranes were first blocked for 1 h in Odyssey Blocking Buffer (LI-COR Biosciences) diluted 1:1 in distilled water and supplemented with 0.2% Tween 20 (OBB-T), then incubated with the appropriate primary Ab diluted in OBB-T at 4 °C overnight under gentle agitation^27^. The membranes were then incubated with an IR Dye 680RD or 800CW goat anti-mouse or goat anti-rabbit secondary Ab (LI-COR Biosciences; 1:15,000 dilution in OBB-T) at RT for 2 h. The infrared signal was detected using the Odyssey CLx Detection System (LI-COR Biosciences). The presence of EV markers TSG101 (Abcam #12501, 1:1000), tetraspaninCD81 (Abcam #109201, 1:500), and the absence of potential contaminants GRP94 (Abcam #ab108606, 1:500), apolipoproteins A1 (Invitrogen #701239), and albumin (Abcam, #ab207327, 1:2000), were assessed by western blotting on three representative samples after the EV isolation and purification protocol described above^27^.

### Lipid extraction and sphingolipid content quantification

Sphingolipid extraction and targeted LC–MS/MS analysis were performed as previously described^30,55^. Sphingolipids were assayed in 25 µL of plasma and in EV deriving from 300 µL of plasma. Both EV pellet and plasma were diluted to 100 µL water and after the addition of 850 µL methanol/chloroform mixture (2:1 v/v), samples were incubated overnight in an oscillator bath at 48 °C. Then, to enhance their recovery, alkaline methanolysis was performed by incubation at 37° for 2 h with 75 µL of potassium hydroxide 1 M in methanol. After neutralization with 75 µL of acetic acid 1 M in methanol, samples were evaporated. The residues were dissolved in 100 µL of methanol, centrifuged for 10 min at 13,400 RPM, and withdrawn in a glass-vial. Finally, samples were analyzed by LC Dionex 3000 UltiMate (ThermoFisher Scientific) coupled to a tandem mass spectrometer AB Sciex 3200 QTRAP (AB Sciex). The separation was achieved by reversed-phase chromatography either using BEH C8 100 × 2.1 mm × 1.7 μm (for ceramides, dihydroceramides and sphingomyelins) or Restek Raptor C18 2.1 × 100 mm × 2.7 μm (for sphingoid bases) by mixing eluent A (0.2% formic acid 2 mM ammonium formate water-solution) and eluent B (methanol 0.2% formic acid 1 mM ammonium formate). Quantitative analysis was performed interpolating each peak area of analyte/area IS with calibration curve for each sphingolipid. Additional LC–MS/MS details can be found in the Supplementary materials (Supplementary Tables S3, S4). Sphingolipids concentration in EV were related to 1 mL plasma.

### Statistics

IBM SPSS Statistics 22 (IBM Corp., Armonk, New York, USA), Python 3.5 (library, scikit-learn), and GraphPad PRISM 7.0a (La Jolla, California, USA) were used for analyses.

The distribution of each parameter was assessed by Kolmogorov–Smirnov test. Normally distributed parameters were expressed as mean ± standard deviation (SD) and analyzed by T-student test or ANOVA test with post-hoc Bonferroni test for multiple comparisons. Non-normally distributed parameters were expressed as median [interquartile range] and analysed by Mann–Whitney’s or Kruskal–Wallis’s tests. Categorical variables were compared through chi square or Fisher tests. Correlations were assessed by Pearson’s R test. P-values < 0.05 were considered significant. The analysis of ROC curves was used to assess the AUC and derive the diagnostic performance in STEMI patient discrimination.

Linear discriminant analysis (LDA) was used as strategy for features reduction to build the canonical plot and evaluate the discrimination power of EV sphingolipid composition as signature to classify patients with STEMI. LDA employs linear combinations of variables to maximize the separation between groups by increasing precision estimates with variance reduction^36^. The algorithm computes a set of coefficients for linear combination of each variable to estimate the diagnosis. The axes of the canonical plot are calculated by the LDA from weighted linear combinations of variables included in the model; each patient is indicated by a point. The crosses indicate the means of (canonical 1; canonical 2) for each group, the ellipses include patients with a linear combination coefficient that falls within the mean ± SD.

## Supplementary information

Supplementary Information.
