# Supplementary material for: Sphingolipid composition of circulating extracellular vesicles after myocardial ischemia
Source: Sci Rep. 2020 Sep 30;10:16182. doi: 10.1038/s41598-020-73411-7 (PMC7527456; doi:10.1038/s41598-020-73411-7)
Supplement: Supplementary file 1 — Supplementary Information. [file 41598_2020_73411_MOESM1_ESM.docx]

**SUPPLEMENTARY DATA**

**Sphingolipid composition of circulating extracellular vesicles after myocardial ischemia**

J. Burrello^1*^, V. Biemmi ^1,2*^, M. Dei Cas^3*^, M. Amongero^4^, S. Bolis^1^, E. Lazzarini^1^,

S. Bollini^5^, G. Vassalli^2,6^, R. Paroni^3^, L. Barile^1,2,7^.

(1) Laboratory for Cardiovascular Theranostics, Cardiocentro Ticino Foundation, Lugano, Switzerland.

(2) Faculty of Biomedical Sciences, Università della Svizzera Italiana, Lugano, Switzerland.

(3) Department of Health Sciences, Università degli Studi di Milano, Milan, Italy.

(4) Department of Mathematical Sciences G. L. Lagrange, Polytechnic University of Torino, Italy.

(5) Regenerative Medicine Laboratory, Dept. of Experimental Medicine (DIMES), University of Genova, Italy. (6) Laboratory of Cellular and Molecular Cardiology, Cardiocentro Ticino Foundation, Lugano, Switzerland. (7) Institute of Life Science, Scuola Superiore Sant’Anna, Pisa, Italy.

*Contributed equally and should be considered as joint first authors.

**SUMMARY**

Supplementary Figure 1 – EV markers assessed by Western Blot (uncropped membranes)

Supplementary Figure 2 – EV contaminants in patients with STEMI vs. healthy controls

Supplementary Figure 3 – Evaluation of EV origin

Supplementary Figure 4 – Ceramides, Dihydroceramides, and Sphingomyelins carried in plasma

Supplementary Figure 5 – Correlation between EV contaminants and Sphingolipids

Supplementary Table 1 – EV characterization by nanoparticle tracking analysis

Supplementary Table 2 – EV surface antigens assessed by Flow Cytometry

Supplementary Table 3 – LC/MS-MS analysis: Ceramides, Dihydroceramides, and Sphingomyelins

Supplementary Table 4 – LC/MS-MS conditions for the analysis of sphingoid bases

Supplementary Table 5 – Ceramides, Dihydroceramides, and Sphingomyelins carried by EV

Supplementary Table 6 – Ceramides, Dihydroceramides, and Sphingomyelins in plasma

Supplementary Table 7 – Correlations with clinical parameters

Supplementary Table 8 – Sphingolipid composition in EV *versus* plasma

Supplementary Table 9 – Correlation of ApoA1 and Albumin with Sphingolipids Levels

Supplementary Table 10 – Comparison of Sphingolipids in STEMI patients after stratification for fasting conditions

**Supplementary Figure 1 – EV markers assessed by Western Blot (uncropped membranes)**

**
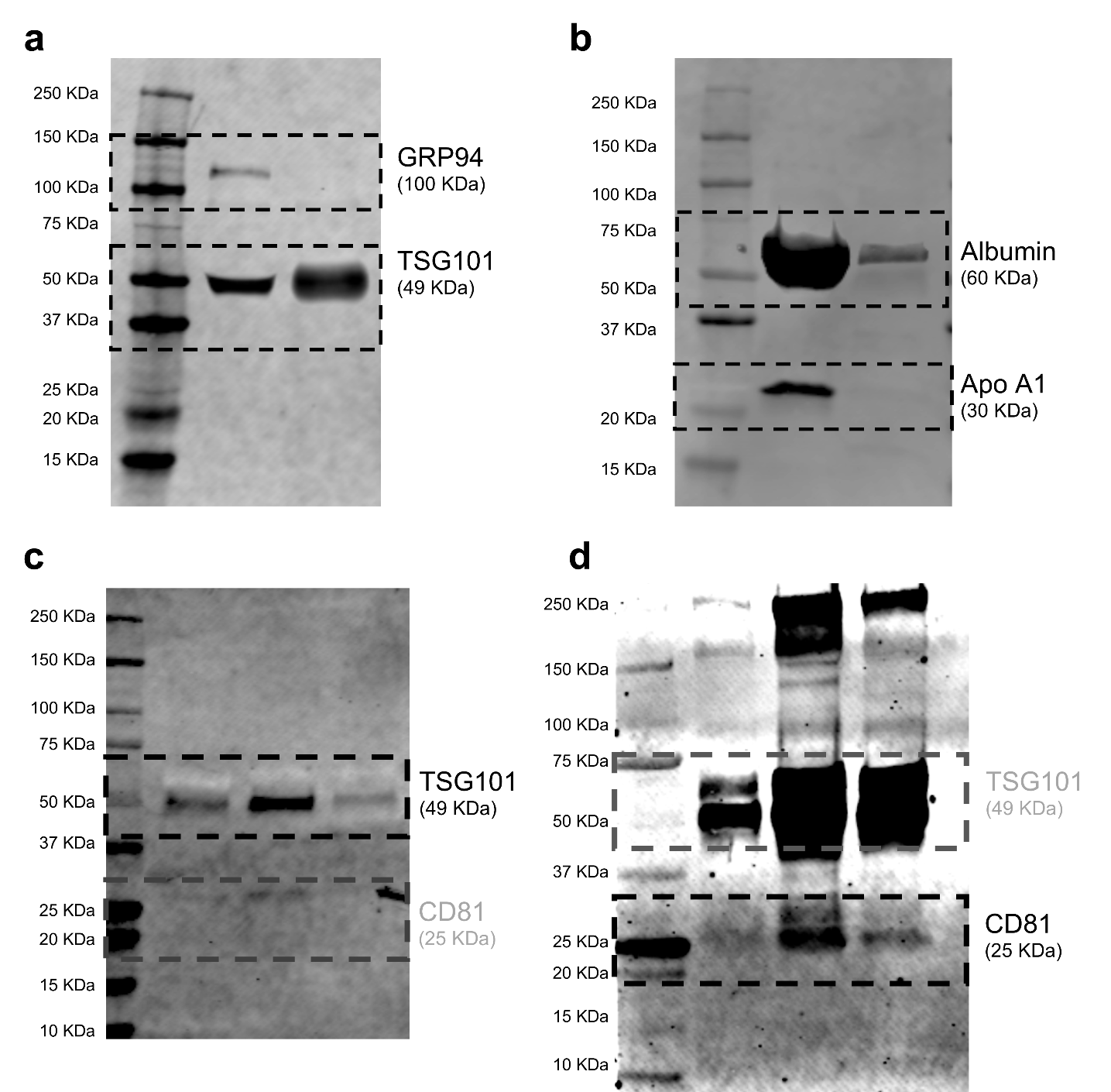
**

Uncropped representative images for western blot analysis reported in figure 1 (GRP94, TSG101, Albumin, and apolipoprotein A1 - panels **a** and **b**) and figure 2 (TSG101 and CD81 – panels **c** and **d**). Black dashed boxes indicate the crop.

**Supplementary Figure 2 – EV contaminants in patients with STEMI *vs.* controls**

**
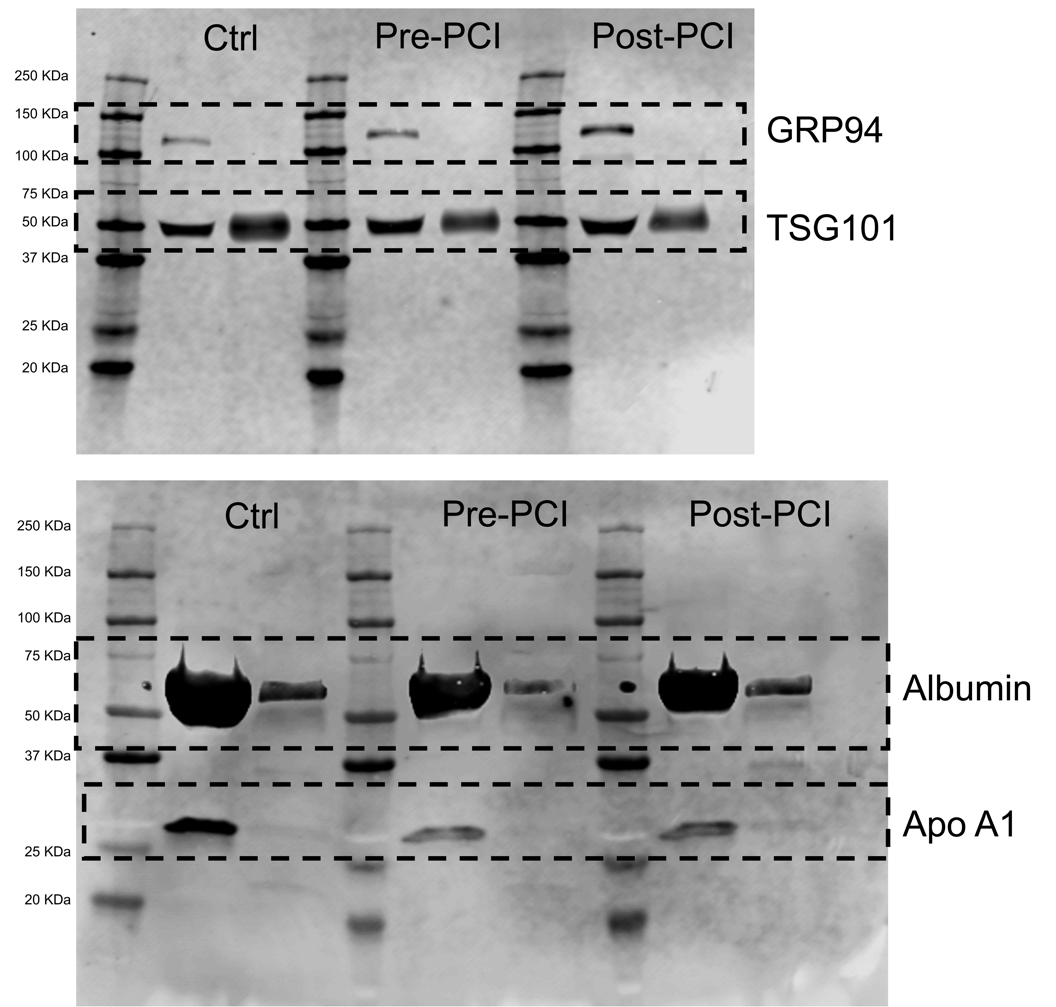
**

Immunoblots for TSG101 and potential EV contaminants (Albumin, Apolipoprotein A1, GRP94) in plasma and plasma derived EV from patients with STEMI before and after PCI as compared to healthy controls.

**Supplementary Figure 3 – Evaluation of EV origin**

**
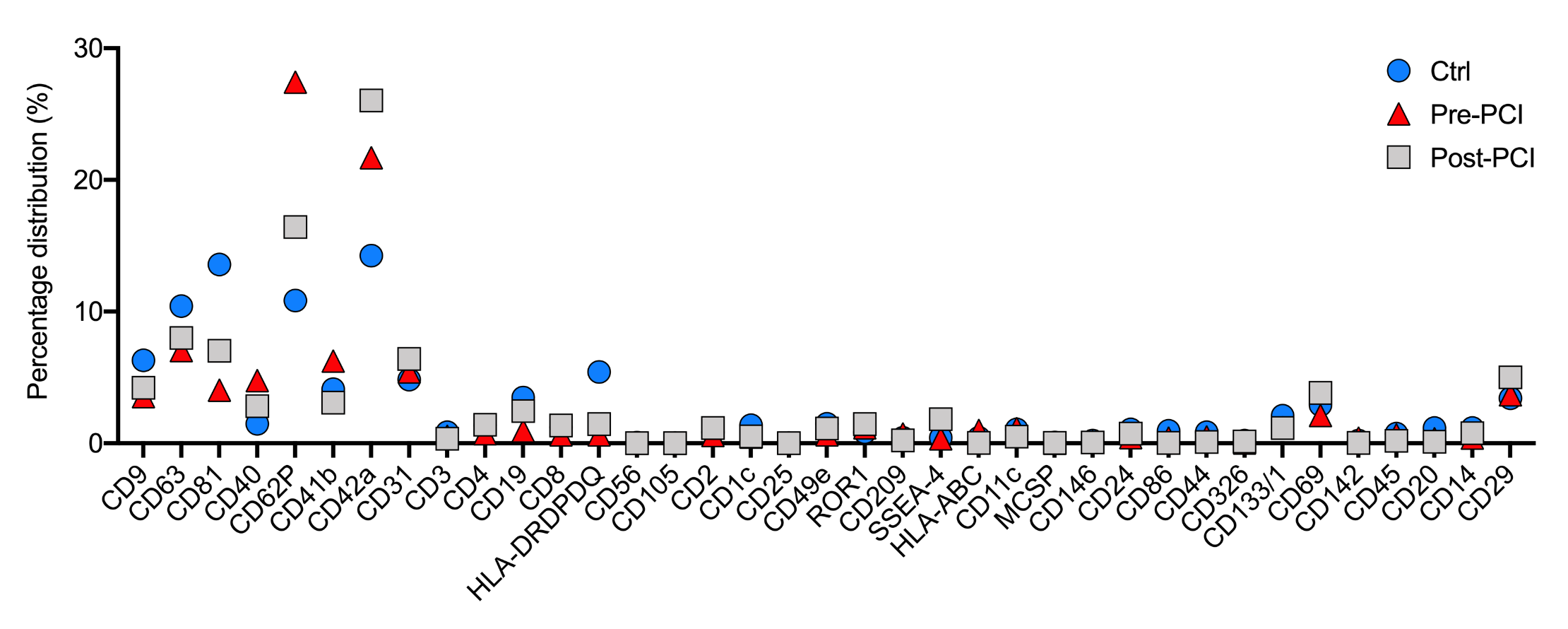
**

EV origin was investigated by flow cytometry for 37 EV antigens commonly expressed on EV surface. Tetraspanins CD9, CD63 and CD81 (EV specific markers) were expressed in all samples. CD40, CD62p, CD41b, CD42a, and CD31 were differentially expressed in patients with STEMI at pre-PCI evaluation as compared to controls and post-PCI patients (see also Table S2).

**Supplementary Figure 4 – Ceramides, Dihydroceramides, and Sphingomyelins in plasma**

**
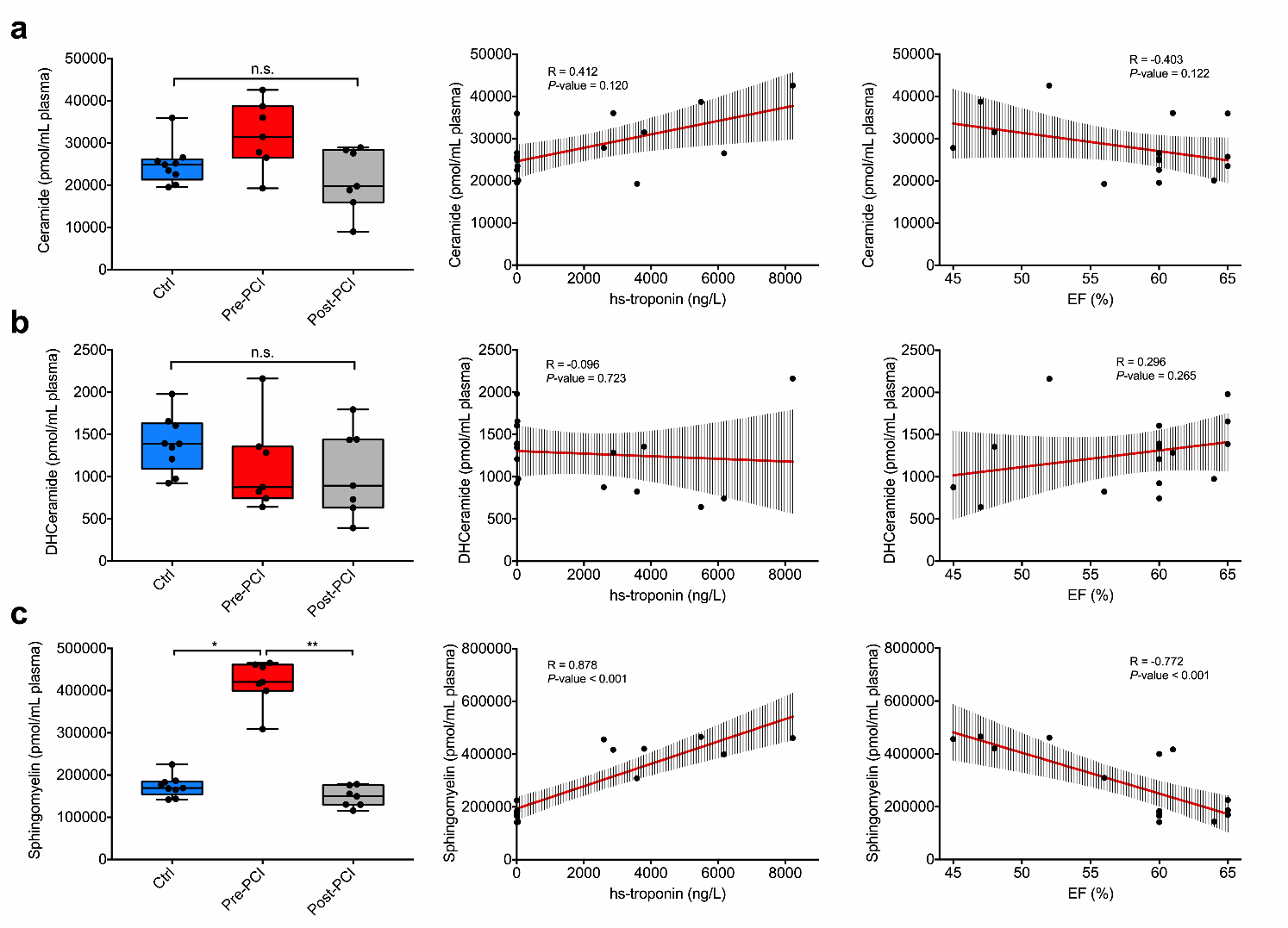
**

Plasma concentration of ceramides, dihydroceramides, and sphingomyelins in patients with a diagnosis of STEMI (n=7) before PCI and after 24 hours from reperfusion, as compared to controls (Ctrl; n=9; left column). Correlations are shown for total ceramides, dihydroceramides, and sphingomyelins to the peak of hs-troponin (ng/L; middle column) and left ventricular ejection fraction at echocardiography at 24 hours after reperfusion (LVEF, %; right column). Regression lines and 95% confidence intervals are shown. Data and statistical analysis: see Tables S5-S6. **p*<0.05; ** *p*<0.01.

**Supplementary Figure 5 – Correlation between EV contaminants and Sphingolipids**

**
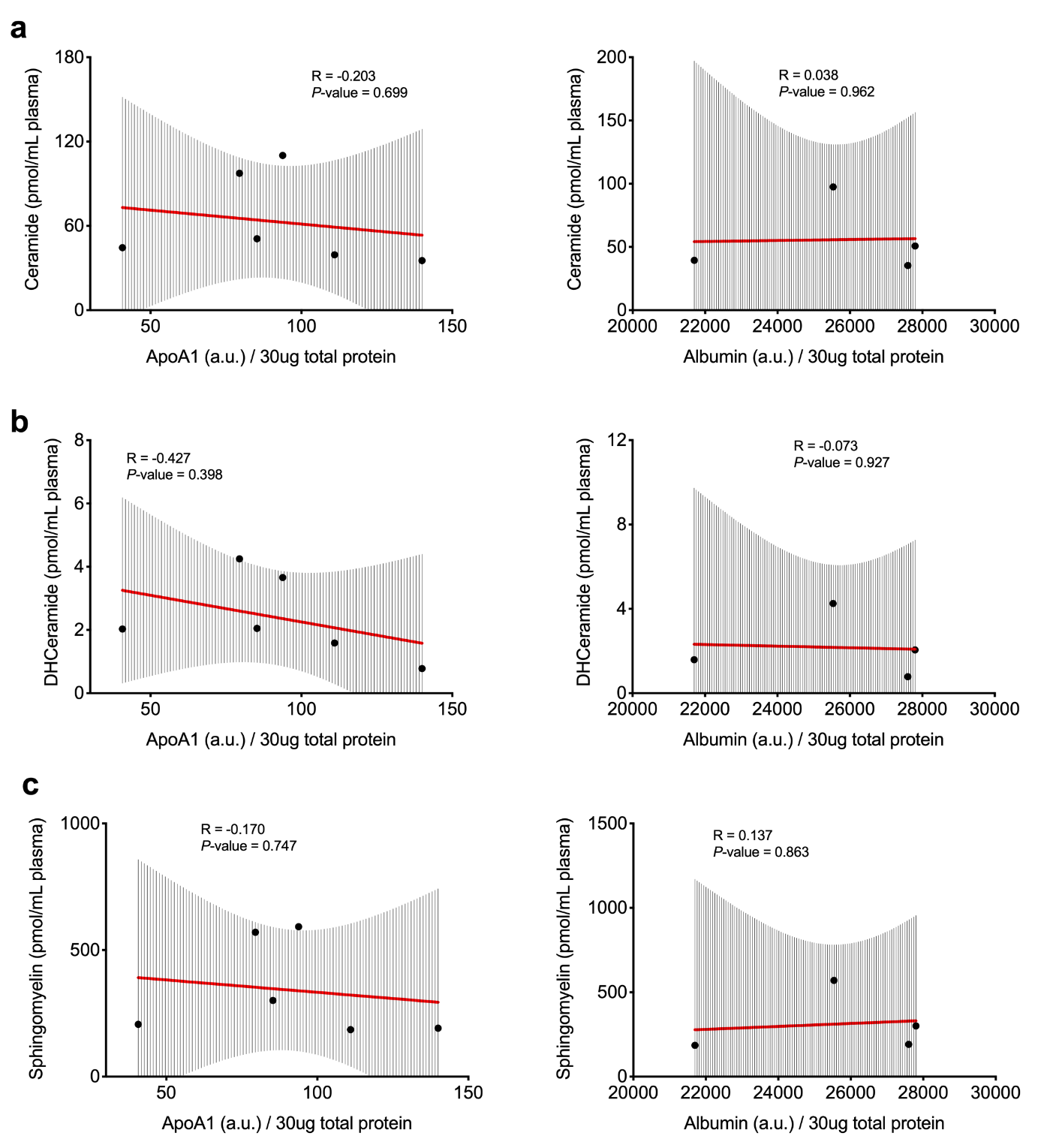
**

Correlations of total ceramides, dihydroceramides, and sphingomyelins to the levels of Apolipoprotein A1 (arbitrary unit, assessed by western blotting; left column) and of Albumin (arbitrary unit, assessed by western blotting; right column). Regression lines and 95% confidence intervals are shown. Data and statistical analysis: see Table S9.

**Supplementary Table 1 – EV characterization by NTA and flow cytometry**

| **Variable** | **CTRL**  [n=9] | **Pre-PCI**  [n=7] | **Post-PCI**  [n=7] | **Overall**  ***P-*value** | **Pairwise Comparisons** | | |
| --- | --- | --- | --- | --- | --- | --- | --- |
|  |  |  |  |  | **Ctrl vs.**  **Pre-PCI** | **Ctrl vs.**  **Post-PCI** | **Pre-PCI vs.**  **Post-PCI** |
| Diameter (nm) | 149 [129-175] | 182 [166-192] | 168 [150-185] | **0.038** | **0.033** | 0.527 | 0.784 |
| EV concentration  (n/mL plasma) | 1.56E11 [1.09E11-3.25E11] | 9.52E11 [7.60E11-3.11E12] | 4.88E11 [2.62E11-6.61E11] | **<0.001** | **0.003** | 0.278 | **0.036** |
| C9 (MFI; %) | 8.81  [4.90-14.86] | 18.16  [17.10-25.67] | 4.64  [3.30-14.11] | **0.041** | **0.048** | 1.000 | 0.054 |
| C63 (MFI; %) | 13.42  [8.61-29.03] | 27.69  [21.31-67.88] | 9.86  [5.51-19.22] | **0.024** | **0.046** | 1.000 | **0.025** |
| C81 (MFI; %) | 20.14  [8.66-39.96] | 21.74  [13.41-39.87] | 10.06  [6.67-23.83] | 0.294 | N.A. | N.A. | N.A. |
| TSG101 (a.u.) | 280.2  [220.7-461.5] | 1284.2  [946.4-2482.0] | 967.6  [385.3-1210.8] | **0.037** | **0.032** | 0.350 | 0.980 |
| CD81 (a.u.) | 137.2  [56.2-183.5] | 287.1  [158.1-624.8] | 126.6  [73.8-191.9] | 0.167 | N.A. | N.A. | N.A. |

Data from Nanoparticle Tracking Analysis, Flow Cytometry and Western Blot. Patients with ST-segment elevation myocardial infarction (STEMI), were compared to healthy controls (Ctrl). For STEMI patients, samples were collected before primary coronary angioplasty (Pre-PCI) and after 24 hours from reperfusion (Post-PCI). EV (Extracellular Vesicle), MFI (Median Fluorescence Intensity), a.u. (arbitrary unit). Data are expressed as median and interquartile range. *P* value < 0.05 was considered significant and indicated by bold characters.

**Supplementary Table 2 – EV surface antigens by Flow Cytometry**

| **MFI (%)** | **CTRL**  [n=9] | **Pre-PCI**  [n=7] | **Post-PCI**  [n=7] | **Overall**  ***P-*value** | **Pairwise Comparisons** | | |
| --- | --- | --- | --- | --- | --- | --- | --- |
|  |  |  |  |  | **Ctrl vs.**  **Pre-PCI** | **Ctrl vs.**  **Post-PCI** | **Pre-PCI vs.**  **Post-PCI** |
| CD3 | 8.0  [4.8-25.5] | 11.0  [3.0-21.0] | 5.33  [1.0-15.5] | 0.742 | N.A. | N.A. | N.A. |
| CD4 | 13.1  [11.4-67.0] | 15.0  [6.3-30.0] | 22.0  [14.0-38.4] | 0.614 | N.A. | N.A. | N.A. |
| CD19 | 33.0  [17.9-37.5] | 19.0  [13.0-31.0] | 38.0  [32.0-44.8] | 0.148 | N.A. | N.A. | N.A. |
| CD8 | 11.5  [6.8-27.8] | 13.0  [8.0-18.0] | 21.0  [10.3-28.0] | 0.489 | N.A. | N.A. | N.A. |
| HLA-II | 51.5  [14.6-110.7] | 13.0  [8.0-38.3] | 22.6  [7.4-38.0] | 0.279 | N.A. | N.A. | N.A. |
| CD56 | 0.3  [0.0-29.9] | 0.0  [0.0-5.3] | 0.0  [0.0-0.3] | 0.513 | N.A. | N.A. | N.A. |
| CD105 | 0.0  [0.0-22.4] | 0.0  [0.0-66.5] | 0.0  [0.0-24.0] | 0.873 | N.A. | N.A. | N.A. |
| CD2 | 8.0  [6.5-26.8] | 12.0  [5.0-26.0] | 17.9  [4.0-30.0] | 0.891 | N.A. | N.A. | N.A. |
| CD1c | 13.0  [7.5-25.3] | 9.4  [4.0-31.0] | 8.3  [0.4-25.0] | 0.659 | N.A. | N.A. | N.A. |
| CD25 | 0.1  [0.1-7.8] | 0.4  [0.2-11.1] | 0.0  [0.0-0.5] | 0.345 | N.A. | N.A. | N.A. |
| CD49e | 14.0  [8.0-16.8] | 13.0  [11.0-22.3] | 17.5  [8.7-20.0] | 0.710 | N.A. | N.A. | N.A. |
| ROR1 | 8.0  [1.1-51.7] | 24.0  [3.0-32.7] | 22.8  [0.5-27.0] | 0.870 | N.A. | N.A. | N.A. |
| CD209 | 4.0  [1.4-8.6] | 14.5  [4.0-27.0] | 3.4  [0.3-8.0] | 0.200 | N.A. | N.A. | N.A. |
| CD9 | 59.8  [38.2-71.5] | 71.8  [55.0-93.2] | 65.7  [61.0-84.8] | 0.224 | N.A. | N.A. | N.A. |
| SSEA-4 | 4.0  [0.4-67.4] | 7.0  [0.1-22.0] | 28.3  [19.2-42.2] | 0.138 | N.A. | N.A. | N.A. |
| HLA-I | 4.0  [0.2-76.5] | 20.0  [3.0-34.9] | 0.5  [0.0-17.0] | 0.352 | N.A. | N.A. | N.A. |
| CD63 | 99.0  [61.4-129.6] | 143.0  [114.0-159.0] | 124.7  [100.9-164.6] | 0.094 | N.A. | N.A. | N.A. |
| CD40 | 14.0  [12.5-55.5] | 96.8  [68.0-142.3] | 44.0  [10.4-67.4] | **0.008** | **0.011** | 1.000 | **0.044** |
| CD62P | 103.0  [56.4-221.0] | 557.0  [255.3-896.0] | 256.2  [99.4-374.0] | **0.010** | **0.008** | 0.278 | 0.622 |
| CD11c | 10.0  [7.9-43.0] | 22.8  [7.0-34.0] | 7.7  [1.0-33.5] | 0.752 | N.A. | N.A. | N.A. |
| CD81 | 129.0  [81.0-196.0] | 82.0  [69.0-106.1] | 109.6  [84.3-114.3] | 0.053 | N.A. | N.A. | N.A. |
| MCSP | 0.6  [0.3-10.1] | 0.1  [0.0-9.6] | 0.3  [0.05.1] | 0.641 | N.A. | N.A. | N.A. |
| CD146 | 2.0  [0.3-11.8] | 2.7  [1.0-9.7] | 1.2  [0.6-8.0] | 0.891 | N.A. | N.A. | N.A. |
| CD41b | 39.0  [25.3-96.7] | 127.0  [87.0-330.0] | 48.0  [36.2-89.0] | **0.035** | **0.044** | 1.000 | 0.127 |
| CD42a | 135.4  [100.5-207.6] | 440.2  [342.0-981.0] | 406.0  [108.5-494.0] | **0.017** | **0.016** | 0.208 | 1.000 |
| CD24 | 10.0  [2.6-26.8] | 9.0  [1.0-39.0] | 11.5  [2.3-22.1] | 0.913 | N.A. | N.A. | N.A. |
| CD86 | 9.0  [2.0-53.8] | 8.0  [0.1-18.5] | 0.5  [0.0-20.0] | 0.627 | N.A. | N.A. | N.A. |
| CD44 | 8.0  [1.6-10.0] | 10.0  [4.0-22.3] | 1.5  [0.3-8.0] | 0.214 | N.A. | N.A. | N.A. |
| CD326 | 1.9  [0.3-23.9] | 0.5  [0.0-7.6] | 2.0  [0.1-15.0] | 0.493 | N.A. | N.A. | N.A. |
| CD133/1 | 20.0  [8.2-69.1] | 24.1  [4.0-68.0] | 17.9  [0.5-28.8] | 0.739 | N.A. | N.A. | N.A. |
| CD29 | 32.4  [20.0-65.0] | 75.0  [64.0-229.4] | 78.0  [24.8-99.9] | 0.065 | N.A. | N.A. | N.A. |
| CD69 | 27.6  [10.6-43.9] | 43.0  [20.0-53.0] | 59.6  [21.0-64.0] | 0.493 | N.A. | N.A. | N.A. |
| CD142 | 2.0  [0.3-18.2] | 8.0  [0.0-30.6] | 0.5  [0.0-31.0] | 0.915 | N.A. | N.A. | N.A. |
| CD45 | 7.0  [0.3-32.1] | 13.2  [3.0-27.0] | 3.0  [0.0-11.5] | 0.571 | N.A. | N.A. | N.A. |
| CD31 | 46.0  [33.1-72.5] | 110.0  [95.0-117.0] | 100.0  [77.8-127.0] | **0.002** | **0.007** | **0.010** | 1.000 |
| CD20 | 11.0  [0.4-33.2] | 7.0  [3.0-14.9] | 1.7  [0.4-9.8] | 0.617 | N.A. | N.A. | N.A. |
| CD14 | 11.0  [3.5-23.2] | 8.0  [4.3-15.0] | 12.1  [4.0-15.0] | 0.928 | N.A. | N.A. | N.A. |

Data from flow cytometry analyses on median fluorescence intensity (MFI; %) for the expression of 37 EV antigens commonly present on EV surface. Data are expressed as median and interquartile range. *P* value < 0.05 was considered significant and indicated by bold characters.

**Supplementary Table 3 – LC/MS-MS analysis: Ceramides, Dihydroceramides, and Sphingomyelins**

| **Analytical column** | ACQUITY UPLC BEH C8 1.7μm 2.1x100mm | | |
| --- | --- | --- | --- |
| **Phase A** | 0.2% formic acid 2 mM ammonium formate water-solution | | |
| **Phase B** | methanol 0.2% formic acid 1 mM ammonium formate | | |
| **LC gradient** (%B) | 0-3 min (80-90%), 3-6 min (90%), 6-15 min (90-99%), 15-18 min (99%), 18-20 min (99-80%), 20-24 (80%) | | |
| **Flow** (mL/min) | 0.3 | | |
| **Column temperature** (°C) | 30 | | |
| **Analytes** | **MS/MS** (m/z) | **DP** (eV) | **CE** (eV) |
| Cer 12:0 (IS)^1^ | 482.7 > 264.4 | 40 | 29.0 |
| Cer 14:0 | 510.7 > 264.4 | 40 | 29.5 |
| Cer 16:0 | 538.8 > 264.4 | 40 | 32.5 |
| Cer 18:1 | 566.8 > 264.4 | 40 | 34.5 |
| Cer 18:0 | 564.8 > 264.4 | 40 | 35.5 |
| Cer 20:0 | 594.8 > 264.4 | 40 | 36.0 |
| Cer 22:0 | 622.9 > 264.4 | 40 | 37.5 |
| Cer 24:1 | 650.9 > 264.4 | 40 | 41.5 |
| Cer 24:0 | 648.9 > 264.4 | 40 | 38.5 |
| DHCer 16:0 | 540.4 > 266.4 | 40 | 33.0 |
| DHCer 18:1 | 568.5 > 266.4 | 40 | 35.0 |
| DHCer 18:0 | 566.5 > 266.4 | 40 | 35.0 |
| DHCer 24:1 | 652.5 > 266.4 | 40 | 40.0 |
| DHCer 24:0 | 650.5 > 266.4 | 40 | 38.5 |
| Sm 12:0 (IS)^1^ | 649.6 > 184.1 | 40 | 50 |
| Sm 16:0 | 705.6 > 184.1 | 40 | 50 |
| Sm 18:0 | 733.6 > 184.1 | 40 | 50 |
| Sm 18:1 | 731.6 > 184.1 | 40 | 50 |
| Sm 24:0 | 817.6 > 184.1 | 40 | 50 |
| Sm 24:1 | 815.6 > 184.1 | 40 | 50 |

**^1^** IS: internal standard

**Supplementary Table 4 – LC/MS-MS conditions for the analysis of sphingoid bases**

| **Analytical column** | Restek Raptor C18 2.7um 2.1x100 mm | | |
| --- | --- | --- | --- |
| **Phase A** | 0.2% formic acid 2 mM ammonium formate water-solution | | |
| **Phase B** | methanol 0.2% formic acid 1 mM ammonium formate | | |
| **LC gradient** (%B) | 0-2 min (20%), 2-4 min (20-99%), 4-7 min (99%), 7-7.5 min (99-20%), 7.5-10 min (20%) | | |
| **Flow** (mL/min) | 0.3 | | |
| **Column temperature** (°C) | 30 | | |
| **Analytes** | **MS/MS** (m/z) | **DP** (eV) | **CE (eV)** |
| Sphinganine d 17:0 (IS) **^1^** | 288.4 > 252.0 | 21 | 20 |
| Sphingosine-1-phosphate | 380.2 > 264.3 | 26 | 21 |

**^1^** IS: internal standard

**Supplementary Table 5 – Ceramides, Dihydroceramides, and Sphingomyelins carried by extracellular vesicles**

| **Variable**  **(pmol/mL plasma)** | **Ctrl**  **(n=9)** | **Pre-PCI**  **(n=7)** | **Post-PCI**  **(n=7)** | **Overall**  ***P-*value** | **Pairwise Comparisons** | | |
| --- | --- | --- | --- | --- | --- | --- | --- |
|  |  |  |  |  | **Ctrl vs.**  **Pre-PCI** | **Ctrl vs.**  **Post-PCI** | **Pre-PCI vs.**  **Post-PCI** |
| **Ceramides** | | | | | | | |
| Cer 14:0 | 0.87 [0.30-1.50] | 0.94 [0.00-1.74] | 1.34 [1.07-2.27] | 0.440 | N.A. | N.A. | N.A. |
| Cer 16:0 | 14.93 [11.16-17.52] | 43.11 [38.77-74.18] | 16.53 [9.67-19.73] | **0.003** | **0.006** | 1.000 | **0.014** |
| Cer 18:1 | N.A. | N.A. | N.A. | N.A. | N.A. | N.A. | N.A. |
| Cer 18:0 | 3.18 [2.47-3.77] | 7.47 [4.83-9.94] | 3.06 [2.41-3.65] | **0.008** | **0.017** | 1.000 | **0.023** |
| Cer 20:0 | 2.24 [1.87-3.01] | 4.07 [3.96-6.48] | 2.41 [1.95-2.70] | **0.043** | **0.048** | 1.000 | 0.100 |
| Cer 22:0 | 3.80 [2.47-3.77] | 12.39 [7.99-16.57] | 4.08 [2.72-4.73] | **0.004** | **0.005** | 1.000 | **0.033** |
| Cer 24:1 | 5.21 [4.36-7.49] | 18.34 [14.77-32.87] | 4.56 [3.87-7.78] | **0.009** | **0.016** | 1.000 | **0.029** |
| Cer 24:0 | 10.58 [9.41-15.36] | 33.59 [20.07-46.60] | 11.98 [10.95-12.64] | **0.005** | **0.007** | 1.000 | **0.025** |
| **Dihydroceramides** | | | | | | | |
| DHCer 16:0 | N.A. | N.A. | N.A. | N.A. | N.A. | N.A. | N.A. |
| DHCer 18:1 | N.A. | N.A. | N.A. | N.A. | N.A. | N.A. | N.A. |
| DHCer 18:0 | N.A. | N.A. | N.A. | N.A. | N.A. | N.A. | N.A. |
| DHCer 24:1 | 0.76 [0.00-0.84] | 1.99 [1.37-3.21] | 0.46 [0.46-1.53] | **0.007** | **0.006** | 1.000 | 0.093 |
| DHCer 24:0 | 1.31 [0.92-1.70] | 2.87 [2.61-3.66] | 0.78 [0.52-1.83] | **0.014** | **0.049** | 1.000 | **0.021** |
| **Sphingomyelins** | | | | | | | |
| Sm 16:0 | 112.73 [92.45-141.15] | 373.89 [329.91-555.97] | 128.50 [80.55-169.75] | **0.005** | **0.007** | 1.000 | **0.035** |
| Sm 18:0 | 25.20 [16.65-31.23] | 73.29 [47.83-107.27] | 24.42 [20.14-33.51] | **0.013** | **0.016** | 1.000 | **0.060** |
| Sm 18:1 | 15.86 [14.55-21.73] | 46.08 [33.19-72.75] | 16.33 [13.11-25.09] | **0.004** | **0.005** | 1.000 | **0.031** |
| Sm 24:0 | 17.19 [12.94-29.11] | 68.83 [46.04-82.41] | 22.36 [16.95-25.60] | **0.034** | **0.034** | 1.000 | 0.192 |
| Sm 24:1 | 28.17 [20-78-44.47] | 78.74 [72.94-125.83] | 27.53 [20.61-55.57] | **0.021** | **0.024** | 1.000 | 0.110 |
| **Total** | | | | | | | |
| Ceramides | 39.69 [32.77-59.05] | 110.13 [97.44-179.85] | 44.47 [34.68-50.81] | **0.004** | **0.007** | 1.000 | **0.015** |
| Dihydroceramides | 1.81 [1.44-2.41] | 5.01 [3.66-6.89] | 2.03 [1.24-2.39] | **0.007** | **0.016** | 1.000 | **0.020** |
| Sphingomyelins | 191.55 [162.85-261.19] | 620.99 [570.19-934.92] | 206.78 [165.48-300.78] | **0.006** | **0.009** | 1000 | **0.028** |
| **Others** |  |  |  |  |  |  |  |
| Sphingosine-1-phosphate | 0.86 [0.00-1.03] | 2.58 [1.05-3.13] | 0.00 [0.00-1.07] | **0.018** | 0.085 | 1.000 | **0.022** |

EV content in ceramides (Cer), dihydroceramides (DHCer), and sphingomyelins (Sm) in patients with a diagnosis of STEMI before PCI and after 24 hours from reperfusion, as compared to controls (Ctrl). Lipid content was expressed in pmol per mL of plasma from which EV were isolated. Data are expressed as median and interquartile range. *P* value *<* 0.05 was considered significant and indicated by bold characters.

**Supplementary Table 6 – Ceramides, Dihydroceramides, and Sphingomyelins in plasma**

| **Variable**  **(pmol/mL plasma)** | **Ctrl**  **(n=9)** | **Pre-PCI**  **(n=7)** | **Post-PCI**  **(n=7)** | **Overall**  ***P-*value** | **Pairwise Comparisons** | | |
| --- | --- | --- | --- | --- | --- | --- | --- |
|  |  |  |  |  | **Ctrl vs.**  **Pre-PCI** | **Ctrl vs.**  **Post-PCI** | **Pre-PCI vs.**  **Post-PCI** |
| **Ceramides** | | | | | | | |
| Cer 14:0 | 273.4 [269.0-362.2] | 136.3 [88.2-152.3] | 237.7 [203.7-204.3] | **<0.001** | **<0.001** | 0.660 | **0.035** |
| Cer 16:0 | 7,519.3 [6,628.2-9,173.1] | 3,511.3 [2,899.0-3,626.5] | 6,535.5 [6,222.9-7,892.2] | **0.001** | **<0.001** | 0.947 | **0.025** |
| Cer 18:1 | 119.5 [98.1-139.2] | 61.8 [46.4-67.0] | 93.1 [82.5-102.8] | **<0.001** | **<0.001** | 0.660 | **0.035** |
| Cer 18:0 | 1,499.1 [1,290.2-1,928.4] | 731.5 [553.6-881.2] | 1,196.8 [1,119.2-1,947.8] | **0.005** | **0.005** | 1.000 | 0.074 |
| Cer 20:0 | 682.5 [566.8-742.7] | 402.0 [286.3-612.6] | 485.6 [430.5-880.0] | **0.034** | **0.028** | 0.766 | 0.504 |
| Cer 22:0 | 1,854.6 [1,727.0-2,017.0] | 2,021.6 [1,490.7-2,778.0] | 1,592.1 [1,087.1-2,751.9] | 0.813 | N.A. | N.A. | N.A. |
| Cer 24:1 | 4,003.0 [3,117.3-4,395.8] | 3.082.9 [2,637.9-3,238.8] | 3,428.0 [2,813.7-4,345.3] | 0.107 | N.A. | N.A. | N.A. |
| Cer 24:0 | 7,980.4 [6,574.9-8,990.3] | 21,215.8 [18,485.1-27,301.6] | 6,669.7 [3,634.3-10,743.3] | **0.001** | **0.006** | 1.000 | **0.002** |
| **Dihydroceramides** | | | | | | | |
| DHCer 16:0 | 230.4 [206.9-276.8] | 191.8 [154.3-225.2] | 163.7 [112.6-254.4] | 0.083 | N.A. | N.A. | N.A. |
| DHCer 18:1 | N.A. | N.A. | N.A. | N.A. | N.A. | N.A. | N.A. |
| DHCer 18:0 | 262.1 [239.0-356.7] | 0.00 [0.00-0.00] | 211.1 [186.8-286.3] | **0.001** | **<0.001** | 1.000 | **0.017** |
| DHCer 24:1 | 320.8 [282.7-425.2] | 113.6 [58.7-230.9] | 272.2 [240.1-336.3] | **0.003** | **0.003** | 1.000 | **0.044** |
| DHCer 24:0 | 489.3 [345.8-654.7] | 627.2 [439.1-1,110.2] | 274.4 [97.2-577.1] | 0.101 | N.A. | N.A. | N.A. |
| **Sphingomyelins** | | | | | | | |
| Sm 16:0 | 86,896.0 [77,735.2-93,542.5] | 192,526 [185,764-211,409] | 80,117.8 [70,729.7-85,354.1] | **0.001** | **0.011** | 0.844 | **0.001** |
| Sm 18:0 | 24,415.8 [21,064.9-28,745.4] | 62,063.0 [48,211.4-70,438.4] | 21,241.2 [16,265.9-27,294.1] | **0.001** | **0.008** | 1.000 | **0.001** |
| Sm 18:1 | 12,911.1 [11,347.9-15,433.7] | 44,713.6 [41,539,9-48,787,7] | 11,466.7 [10,409,2-14,791.0] | **0.001** | **0.006** | 1.000 | **0.001** |
| Sm 24:0 | 21,680.4 [18968.3-25079.6] | 65,211.3 [60,633.3-75,571.5] | 14,210.7 [11,919.6-21,228.5] | **<0.001** | **0.022** | 0.478 | **<0.001** |
| Sm 24:1 | 23,687.3 [20,591.4-28,229.7] | 53,185.0 [44,184.9-54,847.0] | 19,214.0 [17,099.8-23,109.5] | **<0.001** | **0.014** | 0.661 | **<0.001** |
| **Total** | | | | | | | |
| Ceramides | 24,879.8 [21331.7-26,137.8] | 31,483.1 [26,516.0-38,701.4] | 19,754.6 [15,960.8-28,358.4] | 0.066 | N.A. | N.A. | N.A. |
| Dihydroceramides | 1,388.9 [1,092.4-1,631.4] | 876.8 [744.5-1,357.0] | 894.4 [632.7-1,441.5] | 0.235 | N.A. | N.A. | N.A. |
| Sphingomyelins | 169,275 [154,413-184,878] | 420,622 [399,714-461,489] | 149,921 [129,760-176,158] | **<0.001** | **0.014** | 0.661 | **<0.001** |
| **Others** |  |  |  |  |  |  |  |
| Sphingosine-1-P | 482.7 [449.9-562.4] | 762.8 [633.8-778.0] | 389.8 [334.2-444.2] | **0.003** | 0.217 | 0.217 | **0.002** |

Plasma concentration of ceramides (Cer), dihydroceramides (DHCer), and sphingomyelins (Sm) in patients with a diagnosis of STEMI before PCI and after 24 hours from reperfusion, as compared to controls (Ctrl). Lipid content was expressed in pmol per mL of plasma. Data are expressed as median and interquartile range. *P* value *<* 0.05 was considered significant and indicated by bold characters.

**Supplementary Table 7 – Correlations with clinical parameters**

| **Correlation**  **Pearson’s R test**  **(*P-*value)** | **Extracellular vesicles** | | | **Plasma** | | |
| --- | --- | --- | --- | --- | --- | --- |
|  | **hs-troponin (ng/L)** | **LVEF at echo (%)** | **WBC**  **(n/mL)** | **hs-troponin (ng/L)** | **LVEF at echo (%)** | **WBC**  **(n/mL)** |
| **Ceramides** | | | | | | |
| Cer 14:0 | 0.062 (*0.827)* | 0.191 (*0.478)* | -0.419 (*0.107*) | -0.734 (***<0.001***) | 0.664 (***0.005***) | -0.437 (*0.090*) |
| Cer 16:0 | 0.850 (***<0.001****)* | -0.630 (***0.009***) | 0.522 (***0.038***) | -0.784 (***<0.001***) | 0.696 (***0.003***) | -0.497 (*0.051*) |
| Cer 18:1 | N.A. | N.A. | N.A. | -0.749 (***0.001***) | 0.597 (***0.015***) | -0.422 (*0.104*) |
| Cer 18:0 | 0.847 (***<0.001***) | -0.601 (***0.014***) | 0.583 (***0.018***) | -0.586 (***0.017***) | 0.523 (***0.038***) | -0.240 (*0.371*) |
| Cer 20:0 | 0.817 (***0.001***) | -0.613 (***0.012***) | 0.635 (***0.008***) | -0.464 (*0.070*) | 0.288 (*0.280*) | -0.076 (*0.780*) |
| Cer 22:0 | 0.856 (***<0.001***) | -0.610 (***0.012***) | 0.701 (***0.003***) | 0.477 (*0.061*) | -0.223 (*0.406*) | 0.669 (***0.005***) |
| Cer 24:1 | 0.848 (***<0.001***) | -0.606 (***0.013***) | 0.497 (***0.046***) | -0.380 (*0.146*) | 0.278 (*0.296*) | -0.253 (*0.344*) |
| Cer 24:0 | 0.841 (***<0.001***) | -0.596 (***0.015***) | 0.685 (***0.003***) | 0.852 (***<0.001***) | -0.638 (***0.008***) | 0.631 (***0.009***) |
| **Dihydroceramides** | | | | | | |
| DHCer 16:0 | N.A. | N.A. | N.A. | -0.328 (*0.216*) | 0.371 (*0.158*) | -0.443 (*0.087*) |
| DHCer 18:1 | N.A. | N.A. | N.A. | N.A. | N.A. | N.A. |
| DHCer 18:0 | N.A. | N.A. | N.A. | -0.819 (***<0.001***) | 0.694 (***0.003***) | -0.444 (*0.085*) |
| DHCer 24:1 | 0.772 (***0.001***) | -0.371 (*0.157*) | 0.347 (*0.188*) | -0.593 (***0.015***) | 0.484 (*0.058*) | -0.472 (*0.065*) |
| DHCer 24:0 | 0.848 (***<0.001***) | -0.476 (*0.062*) | 0.432 (*0.095*) | 0.531 (***0.034***) | -0.200 (*0.457*) | 0.470 (*0.066*) |
| **Sphingomyelins** | | | | | | |
| Sm 16:0 | 0.833 (***<0.001***) | -0.656 (***0.006***) | 0.560 (***0.024***) | 0.842 (***<0.001***) | -0.764 (***0.001***) | 0.457 (*0.075*) |
| Sm 18:0 | 0.886 (***<0.001***) | -0.636 (***0.008***) | 0.685 (***0.003***) | 0.880 (***<0.001***) | -0.789 (***<0.001***) | 0.618 (***0.011***) |
| Sm 18:1 | 0.896 (***<0.001***) | -0.562 (***0.024***) | 0.619 (***0.011***) | 0.938 (***<0.001***) | -0.721 (***0.002***) | 0.572 (***0.021***) |
| Sm 24:0 | 0.780 (***0.001***) | -0.532 (***0.034***) | 0.638 (***0.008***) | 0.842 (***<0.001***) | -0.691 (***0.003***) | 0.564 (***0.023***) |
| Sm 24:1 | 0.762 (***0.001***) | -0.531 (***0.034***) | 0.427 (*0.099*) | 0.820 (***<0.001***) | -0.806 (***<0.001***) | 0.349 (*0.185*) |
| **Total** | | | | | | |
| Ceramides | 0.859 (***<0.001***) | -0.622 (***0.010***) | 0.591 (***0.016***) | 0.412 (*0.120*) | -0.403 (*0.122*) | 0.482 (*0.062*) |
| Dihydroceramides | 0.630 (***0.001***) | -0.545 (***0.029***) | 0.504 (***0.047***) | -0.096 (*0.723*) | 0.296 (*0.265*) | 0.009 (*0.973*) |
| Sphingomyelins | 0.842 (***<0.001***) | -0.631 (***0.009***) | 0.578 (***0.019***) | 0.878 *(<****0.001***) | -0.772 (***<0.001***) | 0.490 (*0.057*) |
| **Others** | | | | | | |
| Sphingosine-1-P | 0.791 (***<0.001***) | -0.597 (***0.015***) | 0.502 (***0.048***) | 0.673 (***0.004***) | -0.741 (***0.001***) | 0.392 (*0.133*) |

Correlations between the highest level hs-troponin (ng/L) reached by each patient, the left ventricular ejection fraction (LVEF; %) by echocardiography at 24 hours after reperfusion, and white blood cells count (WBC) at presentation to the emergency department, with ceramides, dihydroceramides, and sphingomyelins content quantified in plasma and plasma-derived EV. Analysis was performed on healthy controls and patients with a diagnosis of STEMI at pre-PCI evaluation (n=16). Pearson’s R coefficient and *P*-value were reported for each comparison. The correlation was direct for R > 0, or inverse for R < 0. *P-*value < 0.05 was considered significant and indicated by bold characters.

**Supplementary Table 8 – Sphingolipid composition in EV *versus* plasma**

| **Variable (%)** | **Extracellular Vesicles** | | | **Plasma** | | |
| --- | --- | --- | --- | --- | --- | --- |
|  | **Ctrl**  **(n=9)** | **Pre-PCI**  **(n=7)** | **Post-PCI**  **(n=7)** | **Ctrl**  **(n=9)** | **Pre-PCI**  **(n=7)** | **Post-PCI**  **(n=7)** |
| **Ceramides** | | | | | | |
| Cer 14:0 | 0.36 | 0.12 | 0.51 | 0.14 | 0.03 | 0.14 |
| Cer 16:0 | 6.15 | 5.61 | 6.25 | 3.85 | 0.78 | 3.89 |
| Cer 18:1 | 0.00 | 0.00 | 0.00 | 0.06 | 0.01 | 0.06 |
| Cer 18:0 | 1.31 | 0.97 | 1.16 | 0.77 | 0.16 | 0.71 |
| Cer 20:0 | 0.92 | 0.53 | 0.91 | 0.35 | 0.09 | 0.29 |
| Cer 22:0 | 1.56 | 1.61 | 1.54 | 0.95 | 0.45 | 0.95 |
| Cer 24:1 | 2.15 | 2.39 | 1.73 | 2.05 | 0.68 | 2.04 |
| Cer 24:0 | 4.36 | 4.37 | 4.53 | 4.09 | 4.71 | 3.97 |
| **Dihydroceramides** | | | | | | |
| DHCer 16:0 | 0.00 | 0.00 | 0.00 | 0.12 | 0.04 | 0.10 |
| DHCer 18:1 | 0.00 | 0.00 | 0.00 | 0.00 | 0.00 | 0.00 |
| DHCer 18:0 | 0.00 | 0.00 | 0.00 | 0.13 | 0.00 | 0.13 |
| DHCer 24:1 | 0.31 | 0.26 | 0.17 | 0.16 | 0.03 | 0.16 |
| DHCer 24:0 | 0.54 | 0.37 | 0.30 | 0.25 | 0.14 | 0.16 |
| **Sphingomyelins** | | | | | | |
| Sm 16:0 | 46.41 | 48.67 | 48.61 | 44.49 | 42.73 | 47.75 |
| Sm 18:0 | 10.38 | 9.54 | 9.24 | 12.50 | 13.77 | 12.66 |
| Sm 18:1 | 6.53 | 6.00 | 6.18 | 6.61 | 9.92 | 6.83 |
| Sm 24:0 | 7.08 | 8.96 | 8.46 | 11.10 | 14.47 | 8.47 |
| Sm 24:1 | 11.60 | 10.25 | 10.41 | 12.13 | 11.80 | 11.45 |
| **Others** | | | | | | |
| Sphingosine-1-P | 0.35 | 0.34 | 0.00 | 0.25 | 0.17 | 0.23 |

Sphingolipid percentage composition of extracellular vesicles as compared to plasma, in patients with a diagnosis of STEMI before PCI and after 24 hours from reperfusion, as compared to controls (Ctrl). These percentage were calculated from values in Table S5-S6.

**Supplementary Table 9 – Correlation of ApoA1 and Albumin with Sphingolipids Levels**

| **Correlation**  **Pearson’s R test**  **(*P-*value)** | **ApoA1 (a.u.)** | **Albumin (a.u.)** |
| --- | --- | --- |
| Cer 14:0 | -0.435 *(0.388)* | 0.330 *(0.670)* |
| Cer 16:0 | -0.257 *(0.623)* | 0.050 *(0.950)* |
| Cer 18:1 | N.A. | N.A. |
| Cer 18:0 | -0.148 *(0.780)* | -0.048 *(0.952)* |
| Cer 20:0 | -0.108 *(0.838)* | -0.233 *(0.767)* |
| Cer 22:0 | -0.194 *(0.713)* | -0.084 *(0.916)* |
| Cer 24:1 | -0.107 *(0.841)* | 0.028 *(0.972)* |
| Cer 24:0 | -0.105 *(0.843)* | 0.034 *(0.966)* |
| DHCer 16:0 | N.A. | N.A. |
| DHCer 18:1 | N.A. | N.A. |
| DHCer 18:0 | N.A. | N.A. |
| DHCer 24:1 | -0.223 *(0.671)* | -0.193 *(0.807)* |
| DHCer 24:0 | -0.436 *(0.387)* | 0.022 *(0.978)* |
| Sm 16:0 | -0.225 *(0.668)* | 0.117 *(0.883)* |
| Sm 18:0 | -0.088 *(0.868)* | 0.161 *(0.839)* |
| Sm 18:1 | -0.120 *(0.821)* | 0.261 *(0.739)* |
| Sm 24:0 | -0.057 *(0.914)* | 0.092 *(0.908)* |
| Sm 24:1 | -0.073 *(0.891)* | 0.204 *(0.796)* |
| Ceramides TOT | -0.203 *(0.699)* | 0.038 *(0.962)* |
| Dihydroceramides TOT | -0.427 *(0.398)* | -0.073 *(0.927)* |
| Sphingomyelins TOT | -0.170 *(0.747)* | 0.137 *(0.863)* |
| Sphingosine-1-P | -0.061 *(0.908)* | -0.383 *(0.617)* |

Correlations between levels of apolipoprotein A1 and albumin (assessed by western blot and expressed as arbitrary unit), with ceramides, dihydroceramides, and sphingomyelins quantified in plasma derived EV. Pearson’s R coefficient and *P*-value were reported for each comparison.

**Supplementary Table 10 – Comparison of Sphingolipids in STEMI patients after stratification for fasting conditions**

| **Correlation**  **Pearson’s R test**  **(*P-*value)** | **STEMI patients in FASTING conditions** [n=4] | **STEMI patients NOT in FASTING conditions**  [n=3] | ***P-*value** |
| --- | --- | --- | --- |
| Cer 14:0 | 0.5 [0.0; 1.5] | 1.3 [0.5-1.3] | 0.400 |
| Cer 16:0 | 42.2 [39.4; 78.4] | 54.8 [25.1-54.8] | 1.000 |
| Cer 18:1 | N.A. | N.A. | N.A. |
| Cer 18:0 | 7.0 [5.3-11.1] | 8.7 [3.6-8.7] | 1.000 |
| Cer 20:0 | 4.0 [3.9-7.6] | 5.7 [2.0; 5.7] | 1.000 |
| Cer 22:0 | 10.6 [8.2-20.1] | 13.7 [4.6; 13.7] | 1.000 |
| Cer 24:1 | 17.9 [15.5-29.2] | 22.2 [7.0; 22.2] | 0.857 |
| Cer 24:0 | 31.1 [22.2-59.1] | 40.7 [13.5; 40.7] | 1.000 |
| DHCer 16:0 | N.A. | N.A. | N.A. |
| DHCer 18:1 | N.A. | N.A. | N.A. |
| DHCer 18:0 | N.A. | N.A. | N.A. |
| DHCer 24:1 | 1.6 [0.8-2.9] | 2.1 [2.0-2.1] | 0.229 |
| DHCer 24:0 | 2.9 [2.1-4.2] | 2.9 [2.6-2.9] | 1.000 |
| Sm 16:0 | 367.7 [337.8-650.6] | 452.8 [147.2-452.8] | 1.000 |
| Sm 18:0 | 67.5 [51.3-111.6] | 86.2 [23.9-86.2] | 1.000 |
| Sm 18:1 | 45.6 [36.2-72.6] | 69.2 [20.7-69.2] | 1.000 |
| Sm 24:0 | 66.8 [47.3-100.1] | 68.8 [14.3-68.8] | 0.629 |
| Sm 24:1 | 79.1 [73.9-114.8] | 78.7 [24.0-78.7] | 1.000 |
| Ceramides TOT | 110.6 [100.6-204.0] | 155.8 [57.0-155.8] | 1.000 |
| Dihydroceramides TOT | 4.0 [3.5-7.0] | 5.6 [5.0-5.6] | 0.400 |
| Sphingomyelins TOT | 606.5 [575.6-1,040.6] | 776.8 [230.2-776.8] | 1.000 |
| Sphingosine-1-P | 2.3 [1.3-2.7] | 3.1 [0.0-3.1] | 0.629 |

EV content in ceramides (Cer), dihydroceramides (DHCer), and sphingomyelins (Sm) in patients with a diagnosis of STEMI before PCI stratified according to fasting conditions. Lipid content was expressed in pmol per mL of plasma from which EV were isolated. Data are expressed as median and interquartile range.
